# Prospects and challenges of cancer systems medicine: from genes to disease networks

**DOI:** 10.1093/bib/bbab343

**Published:** 2021-09-01

**Authors:** Mohammad Reza Karimi, Amir Hossein Karimi, Shamsozoha Abolmaali, Mehdi Sadeghi, Ulf Schmitz

**Affiliations:** Department of Cell & Molecular Biology, Semnan University, Semnan, Iran; Department of Cell & Molecular Biology, Semnan University, Semnan, Iran; Department of Cell & Molecular Biology, Semnan University, Semnan, Iran; Department of Cell & Molecular Biology, Semnan University, Semnan, Iran; Department of Molecular & Cell Biology, James Cook University, Townsville, QLD 4811, Australia

**Keywords:** systems biology, transcriptomics, proteomics, metabolomics, proteogenomics, biological networks

## Abstract

It is becoming evident that holistic perspectives toward cancer are crucial in deciphering the overwhelming complexity of tumors. Single-layer analysis of genome-wide data has greatly contributed to our understanding of cellular systems and their perturbations. However, fundamental gaps in our knowledge persist and hamper the design of effective interventions. It is becoming more apparent than ever, that cancer should not only be viewed as a disease of the genome but as a disease of the cellular system. Integrative multilayer approaches are emerging as vigorous assets in our endeavors to achieve systemic views on cancer biology. Herein, we provide a comprehensive review of the approaches, methods and technologies that can serve to achieve systemic perspectives of cancer. We start with genome-wide single-layer approaches of omics analyses of cellular systems and move on to multilayer integrative approaches in which in-depth descriptions of proteogenomics and network-based data analysis are provided. Proteogenomics is a remarkable example of how the integration of multiple levels of information can reduce our blind spots and increase the accuracy and reliability of our interpretations and network-based data analysis is a major approach for data interpretation and a robust scaffold for data integration and modeling. Overall, this review aims to increase cross-field awareness of the approaches and challenges regarding the omics-based study of cancer and to facilitate the necessary shift toward holistic approaches.

## Introduction

According to the world health organization, an estimated number of 10 million patients worldwide succumbed to different types of cancer in 2020 alone. Despite considerable advancements in diagnostics and novel therapeutic approaches following the distilled outcomes of millions of cancer-related studies, many clinical trials do not result in major success [1–3]. This, among other reasons (e.g. implementation issues and technical limitations), can be attributed to the lack of a systemic view toward cancer and its underlying mechanisms. Indeed, the results of the recent WINTHER trial demonstrate the utility of multiomics approaches for the improvement of cancer therapy recommendations [4]. A deeper and holistic perspective of the underlying systemic perturbations during tumor initiation and progression is a prerequisite for designing more targeted a.k.a. personalized interventions.

**Figure 1:**
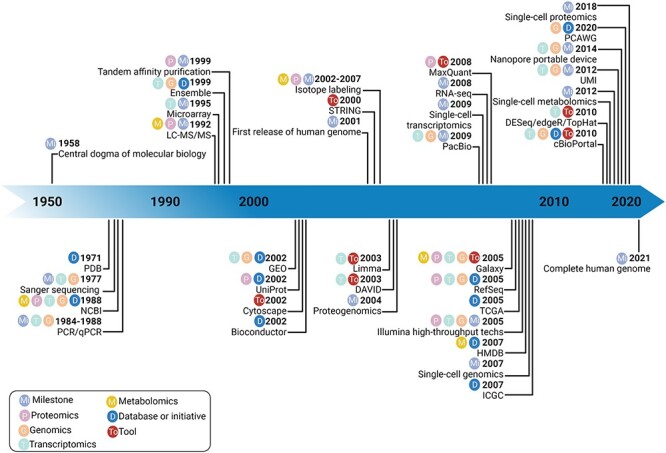
A timeline of some of the major contributions to the field of systems biology.

In cancer investigations, we are facing aberrations in extremely complex systems with enigmatic interplays between altered pathways and extensive multilevel cross-talk. The heterogeneity of subpopulations of malignant cells further contributes to the obscurity of this picture. Contrasting with conventional reductionist approaches, the field of systems biology has emerged and laid foundations for holistic investigation of biological units and mathematical modeling of molecular and cellular interplays for comprehensible exploration of biological systems [5] (refer to Figure 1 for a timeline of some of the major contributions to the field of systems biology). Fueled by genome-wide technologies and bioinformatics advancements, systems biology is establishing itself as the only reasonable approach for dissecting the complexity of tumors, identifying core components of these perturbed systems and recognizing the vulnerabilities of specific tumors for effective patient stratification and precise interventions.

Achieving a holistic picture of cancer demands cooperation between multiple areas of research, magnification of the links between layers of information and robust approaches for effective integration of the heterogeneous data. Hence, there is an increasing need for the research community to move beyond single-layer omics analysis of cancer and take advantage of the value added by integrating multiple omics layers. Here, we review current approaches, methods and technologies that can serve to achieve a systemic perspective of cancer. We start with genome-wide single-layer approaches and move on to multilayer integrative approaches with a focus on a systems biology perspective throughout the work. In each section, an overview of the importance of each respective approach in cancer research is presented. Then, a general framework, based on the current best practices of the field or novel and promising methods, is provided. In that context, we highlight methods that require minimal computational skill and discuss outstanding challenges and future perspectives. It should be noted that while the approaches and technologies discussed in this review are presented in the context of cancer research, many of them are also applicable to fields other than oncology. The review is concluded with multiple representative examples of what these approaches have already contributed to the field of oncology. Overall, we aim to increase cross-field awareness of the approaches and challenges regarding the omics-based study of cancer for both research and medical communities in order to facilitate the necessary shift toward more holistic approaches.

**Figure 2:**
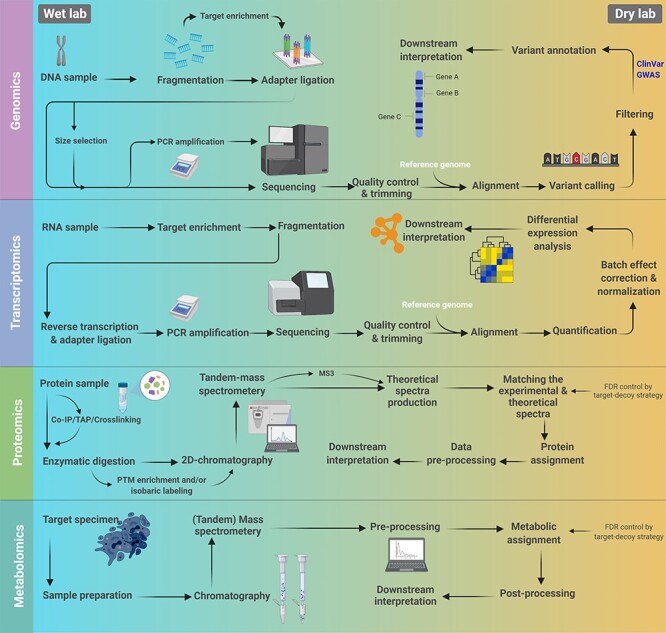
General workflows for different omics studies. The wet lab and computational procedures are distinguished by different background colors.

## Single-layer approaches

High-throughput technologies capable of generating comprehensive data that encompass all the molecular components at a particular level are the main arteries of systems-level studies in cancer. Genomics, transcriptomics, proteomics and metabolomics are the four major approaches currently implemented using various technologies and comprehensive data analysis methods (Figure 2). These approaches and related technologies as well as analysis pipelines are discussed in further sections. Importantly, single-layer data analysis has greatly enhanced our understanding of cellular mechanisms and their perturbations and fundamentally contributed to our knowledge of biological systems. However, the purposive study of biological systems requires multilevel approaches that integrate the generated data from different single-layer approaches to achieve a holistic view of cells under normal and disturbed conditions [6] (for a list of relevant researches and their contributions to the field of systems oncology, refer to Supplementary Table S1, see Supplementary Data available online at http://bib.oxfordjournals.org/).

### Genomics: elucidating the genomic landscape of tumors

The process of tumorigenesis begins (and usually progresses) with the occurrence of specific somatic driver mutations, i.e. mutations that confer survival and proliferative advantages to a specific cell lineage [7]. These mutations are accompanied by a higher number of passenger mutations that do not directly contribute to tumorigenesis and cancer progression. Moreover, germline mutations can contribute to cancer predisposition [8]. The main complexity of cancer, however, arises from the lack of a consensus genomic landscape across different cancer types and even among patients stratified under certain criteria. Case-specific combinations of genomic alterations result in a wide variety of perturbations to the cellular system with the overall similar result of tumorigenesis and cancer progression. Indeed, attempts to discover mutational patterns also known as ‘mutational signatures’ across and within tumor types have significantly contributed to our understanding of the etiology of cancer and led to the identification of cellular processes causative for specific cancer types that can serve as targets for therapeutic interventions [9–11]. Hence, it is evident that achieving an appropriate and encompassing perspective toward this complex disorder necessitates the implementation of genomics technologies.

Whole-exome sequencing (WES) is currently the most widely applied technology both in research projects [12, 13] and in second-tier clinical diagnosis (implemented when gene panels are unable to pinpoint the cause of the defect) [14]. WES was developed to specifically capture and sequence all exonic regions of the genome. However, in the last decade, we have learned that large parts of the human genome that were previously referred to as ‘junk DNA’ are biologically active, i.e. translated into functional noncoding RNA [15]. Point mutations and structural variations in noncoding regions can also be cancer drivers, although less frequently compared to coding regions [16]. These findings, and the downwards trend in costs for sequencing, have already ignited the transition from using WES to whole-genome sequencing (WGS) technologies. WGS has the advantage that it can also identify mutations in intergenic regulatory regions and mitochondrial DNA, mutations in promoters, structural variations and viral infections, all of which are associated with different types of cancer. Moreover, the detection of copy number alterations is more effective with WGS [17]. Interestingly, WGS has been shown to be more effective than WES even when targeting coding regions [14].

Overall, current genomic technologies provide a potent vantage point for studying cancer etiology [10], biomarker discovery [18], the prediction of patients’ drug response [19] and more. Recent years have witnessed the emergence of multiple international efforts such as the Pan-Cancer Analysis of Whole-Genomes (PCAWG) [16] where a considerable number of samples across different tumor types have been sequenced and analyzed. Such efforts provide unprecedented opportunities for the identification of mutational patterns across tumor types and the development of diagnostic and therapeutic approaches that are applicable to a wide range of patients.

#### Experimental workflow and data analysis pipeline

The genomics workflow generally starts with random fragmentation of the purified DNA by sonication or enzymatic digestion. Next, these fragments are enriched for target regions (genes of interest for gene panels or exonic regions when performing WES) [20]. The WGS workflow does not include this step. The acquired fragments are then ligated by oligonucleotide adapters that are complementary to the anchors on the flow cell [21]. This is commonly followed by a size selection step where ligated fragments with suitable sizes are purified [22]. Size selection can increase the sensitivity of circulating tumor DNA detection [23]. Nevertheless, selecting for specific size ranges might result in information loss and, therefore, may be skipped depending on the goal of the study. Depending on the utilized method, a PCR amplification step might be required. However, considering that this step is prone to produce biased results, the utilization of a PCR-free method as a cost-efficient and more effective approach is highly recommended [24, 25]. The next step is the sequencing of the prepared library. Illumina short-read technologies are currently the dominant sequencing platforms (for a comprehensive review of different sequencing technologies, refer to [26]). The NovaSeq 6000 sequencing platform is the most recent Illumina whole-genome sequencing technology. With overall results of similar quality for NovaSeq 6000 in comparison to the older Illumina whole-genome sequencing platform (HiSeq X Ten) and considering the substantial reduction in experiment costs [27], NovaSeq 6000 can be considered as the current state-of-the-art technology for whole-genome sequencing.

WGS data preprocessing begins with demultiplexing the sequencing reads using Illumina’s Consensus Assessment of Sequence And Variation (CASAVA) software. Then, the raw reads are aligned against the human reference genome using an aligner tool, some of the most popular of which are BWAmem [28], Bowtie2 [29] and Novoalign (www.novocraft.com/products/novoalign/). Since duplicate reads can occur during sequence amplification and sequencing procedure, a duplicate marking step using tools such as Picard (broadinstitute.github.io/picard), Sambamba [30] or *SAMBLASTER* [31] is required.

In the next step, variant calling is performed. The most popular variant callers for somatic variant identification that have been specifically developed for the analysis of tumor samples include Mutect2 [32], VarScan [33], Strelka2 [34] and SomaticSniper [35]. A comparative study evaluating the somatic single nucleotide variant calling performance of these tools [36] reported a poor consensus among the results of variant callers. Mutect2 was identified as the best performing tool, followed closely by Strelka. Combining the high-confidence results of these methods is also a recommended approach. The study by Cai *et al.* [36] reported that while this approach increases the specificity of the variant calling, it results in a massive reduction of sensitivity. Thus, a combinatory approach should be opted for if higher reliability is desired while if achieving encompassing results is the goal of the study, utilizing Mutect2 or Strelka is a reasonable approach. In addition, the results of a study comparing the somatic variant calling performance of Mutect2 and Strelka2 [37] suggest that while these tools have similar overall performance, Mutect2 performs better when dealing with lower mutation frequencies while Strelka2 is the better choice in the opposite scenario. Germline variant calling requires a different type of algorithm because the study is confined to the sequencing of normal genome [17]. This is most commonly performed using the Genome Analysis Toolkit (GATK) HaplotypeCaller (software.broadinstitute.org/gatk/). Studies indicate inconsistency among the results of different combinations of aligners and variant callers, and hence, considering the intersection of the results of different pipelines is recommended to reduce false positives [24, 38]. However, a recent study suggests that some popular pipelines can produce results comparable to those of a combination of pipelines [39].

The detected variants are next subjected to annotation procedures. Annotations of previously reported alterations can be obtained from data repositories such as COSMIC [40], ClinVar [41] and OMIM [42]. The impact of novel variants with unknown significance can be predicted *in silico* using bioinformatics tools such as MutationTaster [43], SIFT [44], Polyphen [45] and VEP [46]. This is common practice in clinical diagnosis to predict the impact of novel variants before co-segregation and functional confirmation [47]. Moreover, there are algorithms such as CHASM [48] and PrimateAI [49] that are specifically developed to predict functional effects of mutations in the cancer context and distinguish driver mutations from passengers. The results of a recent comprehensive comparative study [50] that assessed 33 algorithms for their performance in predicting functional effects of mutations in cancer reported that cancer-specific algorithms significantly outperformed algorithms developed for general purposes. Furthermore, this study identified CHASM [48], CTAT-cancer [51], DEOGEN2 [52] and PrimateAI [49] as consistently well-performing algorithms. Notably, it was also proposed that incorporation of pathway and network information of the mutated genes in the prediction algorithm contributed to the outstanding performance of DEOGEN2 and thus, this should be considered in future algorithm developments. Anyhow, insignificant variants are filtered out in this step, while significant variants are reported for downstream analysis and interpretation [53].

It is important to mention that there are numerous pipelines using different combinations of tools and computational approaches that attempt to address different challenges encountered in the various steps of this generalized workflow [27, 54]. There are also convenient and comprehensive tools that facilitate the entire computational procedure, requiring minimal computational expertise. An example is the recently developed portable workflow named Sarek [55].

#### Challenges and perspectives

The variant allele frequency (VAF) is used to determine whether variants are heterozygous (variants with ~50% frequency) or homozygous (variants with ~100% frequency). In the cancer context, however, VAF analysis is not as precise because intratumoral heterogeneity and impurity of tumor DNA cause confusing deviations from expected VAFs [21, 27, 56]. The result of these ambiguities is the inability to acquire a picture of intratumoral heterogeneity that is representative of the actual biological phenomenon. Increasing the sequencing depth toward 100x coverage can ameliorate this inconvenience [24]. However, in some cases, achieving a fully representative picture of intratumoral heterogeneity requires impractical coverages of at least one order of magnitude higher than this [57]. A promising approach to tackle this problem, among others, is single-cell sequencing. Single-cell technologies provide researchers with a more accurate and less complex picture of the perturbed system both in the genomics and transcriptomics context [58]. However, single-cell technologies are still under development and a number of critical challenges both in wet lab [59] and dry lab [60] processes remain to be addressed.

The potential of tumor-specific somatic mutation profiling in guiding the administration of therapeutic interventions with precision is enormous [61]. This attracted a lot of attention toward the assessment of mutational landscapes of individuals through minimally invasive approaches such as cell-free DNA (cfDNA) sequencing. Circulating tumor DNA (ctDNA), presumably derived from necrotic and apoptotic tumor cells, comprises a portion of cfDNA in cancer patients, distinguishing them from healthy individuals [62]. Although the clinical efficacy of cfDNA monitoring in the cancer context is yet to be validated through large-scale clinical trials, potential applications of cfDNA screening make it an attractive subject for researchers. These potential applications include postsurgical monitoring for stratification of patients for adjuvant therapy, systemic monitoring of the heterogeneity of the subclones in a metastatic tumor (as opposed to a single-site needle biopsy) for early detection of resistance to therapeutic agents, and early detection of neoplasms in asymptomatic individuals that can result in more effective interventions [63]. A major challenge for ctDNA analysis is that ctDNA VAFs are usually significantly below the detectable threshold of conventional high-throughput technologies. Ultrasensitive high-throughput technologies dedicated to ctDNA analysis such as iDES-enhanced CAPP-Seq have been introduced to ameliorate this shortcoming [64]. However, various challenges persist. These include increased risk of false positives due to clonal hematopoiesis of indeterminate potential (CHIP) or other diseases and introduction of errors during library preparation (e.g. cfDNA degradation, contamination with normal cell lysates, etc.) and sequencing. Therefore, accurate identification of somatic mutations from cfDNA samples remains a daunting task [65]. Digital PCR approaches for ctDNA monitoring with higher sensitivities and lower costs address some of the challenges associated with high-throughput methods but require *a priori* knowledge of the targets and are particularly low in throughput [65]. Altogether, despite the remaining challenges, the analysis of ctDNA as a complement or surrogate to solid tissue specimens remains a valuable option, especially in cases where solid tumor samples are not accessible or sampling is associated with high risks.

Despite the tremendous progress made in recent years, there are still many unresolved questions in cancer genomics. The fact that no driver mutation could be identified for 5% of tumor samples [16] underscores that despite the extensive study of tumor driver genes and mutations, there are still shortcomings in our knowledge bases and/or models of cancer-initiating perturbations. Indeed, after decades of intensive research in cancer biology, the fundamentals of this complex dysfunction are still ambiguous in some areas. For example, the extent to which additional genomic/epigenomic alterations fuel the transition of a benign tumor to a malignant state is still a matter of debate [66]. Furthermore, the study of the genetic risk modifiers despite their potential to enhance our understanding of cancer is limited due to their small effect size [20]. Another important challenge is pinpointing the genomics alterations in high-complexity regions such as centromeres. Long-read sequencing technologies hold the promise of adequately addressing this problem [67]. However, certain drawbacks such as the high rate of errors in sequencing need to be tackled before these technologies would be able to effectively benefit the field.

### Transcriptomics: approaches to decipher the posttranscriptional complexity of tumors

The central dogma of biology describes the transition of information to function [68], from a semistatic genome to the highly dynamic cell. Going from genome to proteome, the complexity increases as additional regulatory layers are introduced, from epigenetic [69] to posttranscriptional [70] and epitranscriptomic regulations [71], to posttranslational modifications [72]. Hence, efforts to understand the complex mechanisms of the cellular system and its perturbations exclusively from a genomic viewpoint would be futile. A widely appreciated approach to enhance our understanding of this complexity is studying the transcriptome [73].

The qualitative and quantitative analysis of transcriptomic information can yield insights into the posttranscriptional dynamics resulting from genetic events, epigenetic regulation as well as regulation within the transcriptome and provide means to predict the proteomics landscape. In cancer, deviations from normal transcriptomes undergo clonal evolution, which in turn results in converged gene expression patterns referred to as the tumor gene signatures [74] that can be utilized in cancer subtyping [75], biomarker discovery [76], etc. The most broadly utilized functional study of the transcriptome is the comparison of expression profiles under different conditions (e.g. normal versus cancer) known as differential gene expression (DGE) analysis [77], e.g. by means of RNA-sequencing. Differential analysis of mRNA profiles can provide valuable information about perturbed signaling cascades and malfunctioning members of the cell system that gave rise to the phenotype under investigation [78]. The study of alternative splicing and novel splicing events [79, 80], variant calling [81, 82] and fusion transcript detection [83] are some of the other applications of RNA-sequencing with particular importance in cancer.

mRNAs, however, do not constitute the only RNA entities with relevance to cancer [84]. It is now evident that a great portion of noncoding DNA is translated to functional noncoding RNAs (ncRNAs) that are involved in almost all the aspects of cellular processes [85]. There are two general categories of ncRNAs: small noncoding RNAs (sncRNAs) that are less than 200 nucleotides in length and long noncoding RNAs (lncRNAs; >200 nucleotides) [86]. sncRNAs are further categorized into a number of RNA types including microRNAs (miRNAs), small nuclear RNAs and piwi-interacting RNAs. MiRNAs are probably the most widely studied form of ncRNAs [87, 88]. With their recognized role as important regulators of many cellular processes, miRNAs are firmly established as essential players in tumorigenesis and cancer progression and have been widely studied as potential biomarkers and therapeutic targets [89–92]. The role of lncRNAs in cancer, however, is a more recent emerging view [93, 94]. LncRNAs exert a variety of biological functions through interaction with a plethora of different types of macromolecules. LncRNAs’ roles in gene expression regulation through interactions with chromatin, protein complex assembly or disassembly and their interplay with mRNAs have been widely studied [95]. Several lines of evidence attribute a role to lncRNAs in the regulation of virtually all of the cancer hallmarks [96, 97]. The vast number of tissue- and cell-specific lncRNAs along with their importance in the regulation of cellular functions underscores their potential for annotated biomarker discovery in cancer diagnosis, prognosis and treatment [98] as well as their potential employment as therapeutic targets [99].

#### Experimental workflow and data analysis pipeline

Illumina short-read sequencing is currently the dominant platform for transcriptomics studies [100]. The process starts with RNA extraction and target RNA enrichment to remove unwanted rRNAs or specifically select for polyadenylated RNAs through oligo-dT incorporation [101]. However, since other RNA types might be of interest, rRNA depletion can provide more encompassing results [102]. In any case, in the next step, the extracted RNA is subjected to fragmentation in order to become compatible with the short-read sequencing technologies. This is usually done through enzymatic digestion or by using divalent cation-containing solutions [102]. Next, reverse transcription is performed. The second strand of the synthesized cDNA is usually tagged with the incorporation of dUTPs. After the adaptor ligation, the tagged cDNAs are subjected to digestion in order to achieve a strand-specific library [103]. The remaining strands are amplified through PCR and are finally sequenced. The required sequencing depth (total number of reads) is determined by the goal of the study and the nature and condition of the sample [104]. While 15 million reads are considered a saturation point for gene expression profiling [77], a minimum of 70 million reads are required for the accurate quantification of alternative splicing events [105]. This general framework can be modified based on the experimental goals and the RNA type under investigation [106]. The use of single-end or paired-end sequencing or enriching for unique reads restricted to the 3′ end for each transcript in order to analyze DGE are examples of such modifications [102]. Another example is to take advantage of unique molecular identifiers (UMIs) to account for the misrepresentation of biological expression differences due to PCR amplification [107].

The next steps are quality control and preprocessing of the acquired reads [104]. To perform DGE analysis, the level of expression for each gene should be measured from RNA-seq reads. For that purpose, the acquired reads are mapped to an annotated genome or transcriptome using tools such as STAR [108], BWA [109] and TopHat2 [110]. Gene expression is then quantified based on the number of reads that have been aligned to each gene using tools such as HTseq-count [111]. Alternatives include methods such as Sailfish [112], Salmon [113] and Kallisto [114], which implement k-mer counts, quasimapping and pseudomapping, respectively. After batch effect correction [115, 116] and data normalization [117], the last step is the actual differential gene expression analysis. While almost all of the popular methods for transcript quantification have been shown to perform equally well [118], the utilized tool to assess differential gene/transcript expression is an influencing factor in this process. Multiple tools (e.g. NOIseq [119], limma+voom [120] and DESeq2 [121]) are known to perform a high-quality DGE analysis and are accepted as standard tools for DGE assessment [122]. Moreover, the usage of a combination of these tools has been suggested as an effective approach [118]. Quality control in multiple steps of the process (RNA quality, raw reads, alignment and quantification) is also highly recommended [123]. Comprehensive quality control tools such as the NGS QC toolkit [124], RSeQC [125] and Qualimap2 [126] are widely applied to fulfill this purpose.

Multiple tools and web services such as IDEAMEX [127] facilitate an integrated DGE analysis for researchers with a minimal computational background. BP4RNAseq [128] is another user-friendly tool that has been recently introduced and can be utilized for a highly facilitated gene expression quantification. There are also multiple tools and pipelines that are not restricted to DGE analysis and can be implemented for a variety of RNA-seq data analysis purposes. RNACocktail [129] is a comprehensive RNA-seq analysis pipeline incorporating a variety of powerful tools for a variety of purposes including RNA variant-calling, RNA editing and RNA fusion detection.

RNA-sequencing is at the forefront of single-cell sequencing technologies [130, 131]. Sensitive full-length transcript sequencing platforms such as MATQ-seq [132] with the ability to capture and sequence ncRNAs herald the arrival of a new level of sequencing capacity. The general workflow for single-cell sequencing is similar to the bulk RNA-sequencing workflow described above [133]. It is indeed possible to perform most of the computational processing steps with the bulk RNA-sequencing methods. However, low levels of starting material coupled with additional technical requirements (such as cell-specific barcoding to be able to demultiplex the resulting data from multiplexed sequencing) and other challenges (such as the possibility of capturing damaged, dead or multiple cells) necessitate the development of computational methods tuned for single-cell analysis [134, 135] (see Table 1 for a list of single-cell RNA-sequencing tools). It should be noted that large-scale comparative studies are required for the assessment of the utility of these tools in comparison with one another and with the tools designed for bulk-RNA sequencing analysis. Indeed, bulk-RNA sequencing analysis tools have been shown to be capable of producing satisfying and, in some cases, superior results compared to that of the tools specifically designed for single-cell RNA-seq [136].

**Table 1:** A list of tools dedicated to single-cell RNA-seq data manipulation and analysis

Name	Implementation	Description	Reference
*Alona*	Web-based	A comprehensive and user-friendly tool that supports quality control, normalization, batch-effect correction, cell type identification, DGE analysis and visualization	[367]
*Census*	R	An algorithm that performs gene expression quantification and differential analysis	[368]
*CMF-Imputation*	MATLAB	A tool that performs the imputation of the dropout events in the expression matrix	[369]
*DoubletFinder*	R	A tool that identifies and removes doublet events using gene expression data	[370]
*DrImpute*	R	An algorithm that sequentially imputes the dropout events	[371]
*MNN*	R	An algorithm that accounts for batch effect noise through detection of mutual nearest neighbors	[372]
*SAVER*	R	A tool for quantification of gene expression in single-cell RNA-seq studies that incorporated unique molecular identifiers	[373]
*Seurat*	R	A comprehensive and highly powerful toolkit designed for single-cell data manipulation and integration	[374]
*Scater*	R	A comprehensive R package capable of performing gene expression quantification, quality control, normalization and visualization	[375]
*SCDE*	R	A Bayesian approach for DGE analysis	[376]
*SCENIC*	R	An algorithm for the identification and analysis of cellular regulatory networks	[377]
*scGEAToolbox*	MATLAB	A user-friendly and comprehensive toolkit that supports batch effect correction, normalization, imputation, feature selection, clustering, trajectory analysis and network construction and can readily be incorporated in customized workflows	[378]

#### Challenges and perspectives

A current challenge in RNA-sequencing is that the reconstruction of full-length RNA molecules from short reads is error-prone [104]. This results in incorrect assignment of reads and misrepresentation of isoform abundances and also makes isoform discovery a challenging task. Long-read technologies, as well as synthetic long-read methods, hold the promise of solving this inconvenience [100]. However, various challenges remain to be addressed. Long-read technologies are particularly low in throughput. This problem in turn would result in a reduced experiment size and low sensitivity of differential expression [100]. Hence, using a long-read technology is not currently recommended for DGE analysis, particularly when the study involves low expression levels. The high error rates and additional costs are prohibitive elements regarding long-read technologies. Moreover, the rigorous requirement to avoid RNA degradation and shearing during sample handling makes the achievement of high-quality samples laborious. However, the combination of short-read with long-read sequencing methods enhances the quality and accuracy of transcript isoform expression analysis. For instance, by combining these technologies and using algorithms for hybrid assembly of short and long reads (hybridSPAdes; [137]), enhanced results for *de novo* transcriptome assembly (e.g. with rnaSPAdes; [138]) can be achieved.

### Proteomics: studying the frontline of phenotype manifestation

Virtually all the regulatory mechanisms governing the central dogma of biology eventually serve to determine the set of expressed proteins, their expression levels and the manner in which they function; the deviations of which from normal status can result in a malfunctioning system and give rise to various disorders such as cancer [139]. Proteins can be considered as frontline agents of phenotype manifestation, and hence, studying proteome-level regulatory mechanisms, such as posttranslational modifications (PTMs), the inherent properties of proteins (e.g. their 3D structures) and protein–protein interaction (PPI) networks, is essential if representative views of the normal and perturbed cellular system are to be achieved. Moreover, the validity of inferring protein abundance from mRNA expression has been questioned due to the lack of consistently strong correlations between mRNA and protein abundance [140], suggesting that the direct assessment of protein abundance is a more reliable source.

All of the categorized hallmarks of cancer are either directly regulated by proteins or are highly affected by them [141]. Proteins function in protein assemblies and highly complex networks. In this context, malfunction in any member of these networks can potentially result in the disruption of the activity of other members of the same network. Therefore, an important goal of proteomics studies, in addition to assessing genome-wide protein expression under various conditions, is to achieve comprehensive and functional models of all the physical protein interactions both in normal and perturbed conditions [142]. Equally important is the study of PTMs. With more than 450 types of PTMs, these modifications regulate protein expression levels and almost all cellular processes, such as immune response, apoptosis, tumorigenesis and cancer progression [143–146]. Exploration of these and other aspects of cell biology from omics data of other levels is either impractical or impossible. Collectively, current proteomics technologies and approaches provide researchers with powerful assets in the quest of achieving a functional view of the cellular system and addressing fundamental questions regarding the biology of cancer as well as discovering biomarkers and actionable therapeutic targets [147, 148].

#### Experimental workflow and data analysis pipeline

Multiple methods have been developed to assess the proteomic landscape of cells and tissues. Targeted and top-down proteomics [149, 150] are two of the established branches of such methods with dedicated software tools and platforms [151–153]. However, data-dependent bottom-up or ‘shotgun’ proteomics through liquid chromatography–tandem mass spectrometry (LC–MS/MS) is currently the *de facto* standard approach for genome-wide proteomics analysis [154]. The workflow for shotgun proteomics is variable and context-dependent. A general workflow based on the current best practices can be presented as follows: after the lysis of the samples, the disulfide bridges of the extracted proteins are disrupted through reduction and alkylation of the cysteine residues. Next, the proteins are subjected to enzymatic digestion through the addition of proteinases (most commonly Lys-C followed by trypsin). One- or two-dimensional chromatography is next applied; the latter is recommended to increase the dynamic range (i.e. to provide the possibility for low-abundance proteins to be identified) [155]. Currently, the most effective approach is to subject the samples to basic reversed-phase chromatography followed by acidic reversed-phase chromatography as the second dimension [156]. There is also the choice between label-free and isobaric labeling [using iTRAQ [157] or tandem mass tags (TMTs, [158])]. Isobaric labeling approaches are recommended due to the provided capacity for multiplexation and the reduction of errors from manual sample handling as well as higher precision in quantification, especially when PTMs are the target of the study [155]. The wet lab procedure is concluded by the acquisition of MS spectra from MS/MS. Orbitrap-based MS/MS is the current standard. It is also possible to add a third stage (MS3) by combining Orbitrap and Ion Trap methods and it has been shown to be effective when facing highly complex samples [159]. For comprehensive and step-by-step workflows for the wet lab procedure, refer to [155, 159].

Although methods exist for *de novo* identification of peptide sequences [160], current approaches still suffer from high error rates. The preferred method is to first prepare a database of all the known protein sequences (comprehensive databases such as UniProt [161] can be exploited for this purpose) and subject them to *in silico* digestion according to the properties of the proteinase enzymes that were utilized during sample preparation. The resulting *in silico*–produced peptides are then assigned theoretical spectra and the experimentally acquired spectra are searched against this database. Each match is scored based on the similarity and the highest-scoring match reveals the identity of each peptide with a certain false discovery rate (FDR). A stringent FDR of 1% is recommended [162]. The recommended approach to control for this FDR is the target-decoy search strategy [163]: a parallel database of incorrect peptides is constructed (usually through reversion of the peptide sequences of the main database). Matches to this database are obviously false positives and, hence, can reveal the FDR based on the utilized filters. Using this method, one can tune the applied filters to achieve a suitable FDR. The identified peptides are then assigned to their respective proteins. Peptides with less than seven residues are usually nonunique and are prone to erroneous protein assignment and, thus, are recommended to be excluded [162].

Proteomics data need to be preprocessed (including normalization, filtering, etc.) before they can be interpreted in a biological context. After preprocessing, the data can be manipulated to yield functional information through a variety of approaches. Differential expression analysis is a common approach with subsequent context-specific analyses such as expression signature discovery and co-expression network analysis.

The general workflow provided here can also be modified in order to customize the study for the analysis of PTMs [164], PPIs and subcellular localization [142]. For the analysis of PPIs, target protein complexes should be isolated from the cell lysate. Co-immunoprecipitation (Co-IP) is a common approach for this purpose [165]. Co-IP involves the attachment of specific antibodies to bait proteins (proteins whose interacting partners are under investigation). These antibody–protein complexes are captured by agarose beads attached to A/G proteins and are ‘pulled-down’ by means of centrifugation. Proteins in tight interaction with the bait proteins are also precipitated in this step and the unbound components of the lysate are discarded. The captured proteins can then be subjected to MS to identify PPIs. Tandem affinity purification (TAP) is a similar approach with enhanced purification that involves tagging the bait protein at its N-terminus by a TAP tag (usually a calmodulin-binding domain followed by a highly specific protease cleavage site followed by an IgG-binding fragment) prior to two steps of purification by centrifugation [166]. The major problem associated with these approaches is their restriction to identify highly stable PPIs. For the identification of more transient interactions in complex biological samples, another method termed cross-linking-MS (XL-MS), which also has the advantage of providing spatial information, is favored [167]. This method is based on covalently binding residues in two proteins through two reactive groups (usually amine-groups due to the prevalence of lysin residues in protein structures) that are connected via a spacer with a finite distance. This limited distance confers a spatial constraint on the residues that can be linked; making the cross-linking possible only between proteins in close proximity (i.e. interacting proteins). As for the PTMs, the mass shift in the peptides due to these modifications is identifiable by LC–MS. However, an additional enrichment step for the peptides with the modification under investigation is required [168]. Various strategies for this enrichment including implementation of immunoaffinity precipitation (using antibodies highly precise for specific types of modification) and chromatography-based approaches (e.g. immobilized metal ion affinity chromatography, metal oxide affinity chromatography, etc.) have been devised. The most suitable approach, however, is dependent on the type of modification under study and the specific physical/chemical properties it confers to the peptides (refer to [168, 169]).

MaxQuant [170] is a popular comprehensive platform that along with Perseus [171] facilitates the entire procedure of shotgun proteomics data analysis. Moreover, dedicated platforms for computational analysis of PTMs and PPIs exist [172, 173]. In addition, a recently developed comprehensive toolkit named ‘Philosopher’ [174] demonstrates a movement toward making these computationally sophisticated methods accessible to a broader community.

The prospective results of the ‘discovery’ shotgun proteomics can be channeled into ‘hypothesis-driven’ targeted proteomics for validation in order to extract actionable and clinically relevant directions from the plethora of information resulted from shotgun proteomics [175]. Targeted proteomics approaches are higher in sensitivity and dynamic range and tackle the problem of irreproducibility associated with shotgun proteomics, which is due to the stochastic nature of precursor ion selection in shotgun approaches. Targeted proteomics is developed based on prior knowledge about the proteins of interest and the selection of signature peptides that specifically represent those proteins. Selected reaction monitoring (SRM) is a widely-used targeted approach. A triple quadrupole instrument is used to filter the target peptides based on their predetermined mass-to-charge ratio, which combined with their elution time can be sufficiently specific. The filtered peptides are subsequently fragmented using collision-induced dissociation and the resulting fragment ions are once more filtered for specific fragments based on a predetermined mass-to-charge ratio. This process is repeated for multiple different fragment ions of each filtered peptide and, hence, peptides are identified and quantified utilizing MS spectra [176]. Parallel reaction monitoring (PRM) is a similar approach, which through the implementation of an Orbitrap or time-of-flight instrument removes the second filtering step by analyzing all the fragment ions simultaneously and provides more accurate results [176].

#### Challenges and perspectives

In spite of the remarkable progress made in proteomics methods in the last decade [147], drawbacks such as the cofragmentation problem [177] still exist and experiment design approaches, as well as computational strategies, are being constantly revised to compensate for these [178]. Overall, reduction in costs and a further increase in the sensitivity of mass spectrometers can be considered as major factors that can enhance the efficiency and accessibility of proteomics analyses [179]. Specific to targeted proteomics, a major drawback of SRM and PRM approaches is that the analysis is restricted to the preselected target proteins. Recent advances in data-independent acquisition methods (particularly SWATH-MS) circumvent the need for repeated measurements for each target protein by allowing posterior querying of the data for the desired peptides while providing multiplexing capacities comparable to shotgun proteomics [180]. However, data-independent acquisition methods lack the sensitivity of SRM and PRM and are therefore inferior to these approaches when dealing with very low-abundant proteins. In addition, SWATH-MS is still facing challenges regarding ease of data analysis [180].

From the clinical perspective, minimally invasive sample collection is critical. Body fluids (e.g. blood, saliva, urine, tears, etc.) are readily available rich sources of biomolecules (e.g. over 12 000 proteins only in plasma) with altering compositions during tumor development, which can be used as tumor and/or stage-specific biomarkers [181]. Proteomics approaches were generally successful in discovering such biomarkers [182, 183]. A major pitfall associated with body fluid biomarker discovery, however, is the massive dynamic range: a handful of enormously abundant proteins mask the presence of lowly abundant molecules of interest. Strategies such as immunodepletion of high-abundance proteins have been devised, which nevertheless face the caveat of information loss due to unspecific bindings to affinity ligands [184]. Nonetheless, the achievements of multiple efforts in recent years underline the possible widespread utilization of these sample types in clinical practice in the future [185, 186].

Single-cell proteomics is a promising prospective approach that is still in its infancy. For single-cell technologies to become a feasible practice in proteomics, advances in both technological and computational aspects are required [187]. Considerable increase in MS sensitivity and the development of specialized tools for the analysis of such data are prerequisites of making single-cell proteomics practical. Nevertheless, various multidisciplinary efforts are already turning the dream of single-cell proteomics into reality [188].

### Metabolomics: exploring the survival strategies of cancer cells

During cancer initiation and progression, cellular systems are reprogrammed to grow and proliferate at exceptionally high rates and to acquire an enhanced capacity for survival under extreme conditions [141]. Clearly, a considerable portion of this reprogramming is dedicated to shaping an altered form of metabolism that is able to meet the massive energy needs and to provide required anabolic precursors for these highly demanding self-centered systems. Indeed, almost every aspect of cellular metabolism is affected during cancer progression [189] and since the metabolic status of a sample can be considered as the ultimate downstream manifestation of the effects of both intrinsic (e.g. genetic) and extrinsic (i.e. environment) factors on the biological system [190], valuable insights can be gathered from the study of the metabolome.

Two core metabolites with altered metabolic pathways in cancer are glucose and glutamine [191]. Excessive glucose fermentation, overexpression of the rate-limiting enzymes of the glycolysis branch pathways, constitutive glucose influx, as well as an increased expression rate of glutamine synthesis are examples of such alterations that cancer cells exploit to provide themselves with modified sources of energy and a large collection of biosynthetic precursors [189]. In addition, cancer cells develop scavenging strategies in order to survive under the commonly encountered nutrient-poor microenvironment. These strategies include autophagy [192], consumption of extracellular proteins through macropinocytosis and subsequent lysosomal degradation of these molecules [193], entosis [194] and phagocytosis [195], as well as induction of fatty acid release from neighboring cells [196]. Cancer cells also highly influence the condition of their microenvironment. The high rate of glucose fermentation results in the accumulation of considerably high levels of extracellular lactate and H^+^, which in turn contribute to angiogenesis, immune response suppression and tumor invasiveness [189]. Since the survival of cancerous cells is highly dependent on this altered metabolic status, the metabolome is an active area of research for the discovery of cancer biomarkers as well as the identification of potential therapeutic targets [197, 198].

The contribution of metabolites to the initiation of signaling cascades and their effect on the epigenetic landscape as well as PTMs are other topics of investigation. Through these investigations, the role of metabolites not only as molecules with altered behavior downstream of cancer initiation and progression but also as etiological agents (i.e. oncometabolites) that contribute to system perturbations is being rapidly established [199]. Further studies of the metabolome in this context have the potential to shed light on novel aspects of cancer biology.

#### Experimental workflow and data analysis pipeline

Due to the inherent chemical homogeneity of the polymers of genome, transcriptome and proteome, it is possible for a single platform to capture a holistic snapshot of each respective layer. However, this does not hold in metabolomics owing to the chemical heterogeneity of different classes of metabolites [200]. Proton nuclear magnetic resonance (1H NMR) and MS-based methods are the most common approaches for metabolomics data acquisition; all of which are associated with various advantages and disadvantages [190].

NMR is highly reproducible, is conveniently quantifiable, requires minimal sample preparation and unlike MS-based approaches is nondestructive [190, 201, 202]. Moreover, it is considered the gold standard method for the elucidation of the metabolite structures [203]. Nevertheless, NMR suffers from low sensitivity and it is only capable of detecting 20–50 metabolites per sample, which is an inadequate number for systems-level analyses [190]. MS-based approaches, on the other hand, possess the advantage of high sensitivity and are widely adopted for untargeted and system-level metabolomics analyses due to their capability to detect 100–1000 metabolites per sample [200, 203]. Gas chromatography-MS (GC–MS) and LC–MS (or LC–MS/MS) are the most commonly used methods for MS-based metabolomics [204]. GC–MS is cost-effective and has the advantage of a virtually automated metabolite identification process. However, it is only applicable to volatile and thermally stable metabolites or those that can be adapted for the process with chemical derivatization [203]. This limits the versatility of GC–MS. In addition, the derivatization process can introduce artifacts and might result in erroneous quantification because of incomplete derivatization [205]. Unlike GC–MS, LC–MS does not require derivatization and with the ability to capture molecules in a wider weight range, it is highly versatile and efficient [190, 203, 204, 206]. While these advantages make LC–MS the most widely applied method in the field, researchers are encouraged to opt for a combination of these approaches to achieve a more comprehensive representation of the metabolic status of the sample [201]. The workflows for all of the above-mentioned approaches are somewhat similar, with nuances and differences in the steps and applied algorithms. However, due to the extensive utility of the LC–MS and LC–MS/MS, these approaches are the main focus of this section.

Unlike NMR, MS-based analysis needs a sample preparation step consisting of protein precipitation and liquid-phase extraction [207]. The higher susceptibility of the metabolome to alter under different conditions in comparison to the other omics layers [208] means that careful experimental design is a requirement to minimize confounding factors. The instruments with high mass-resolving power such as LTQ-Orbitrap and Q-TOF are instruments of choice for systems-level metabolomics. Electrospray ionization (ESI) is the most widely applied ionizing method in order to make the metabolites detectable in LC–MS metabolomics [204, 209]. Of note, the validation of the results of untargeted studies through targeted approaches can increase the reliability of the acquired data [206].

The general computational workflow consists of preprocessing, peak detection or annotation, postprocessing and statistical analysis of the resulting data [210]: after the data are obtained, they should be subjected to the preprocessing procedure in order to enhance comparability and management [190]. Preprocessing usually starts with peak picking, which is the process of detecting the actual informative regions of spectra and removing the background noise. For MS-derived data, a deconvolution step is required to reduce redundancy. Another requirement is the alignment of matching peaks between different samples [211, 212]. A practical and popular approach for peak annotation (i.e. the assignment of the observed peaks to actual metabolites) is to search the data against the existing spectral libraries in a process similar to what has been described in the proteomics section. The desired information for metabolites is acquired by inquiring metabolome databases such as the Human Metabolome Database (HMDB) [213], METLIN [214] and MassBank [215]. It is also possible to implement a target-decoy strategy to control for the FDR. An innovative approach regarding the construction of a decoy database for metabolome studies has been proposed by Wang *et al.* [216], which is performed by violating the octet rule through the addition of extra hydrogen atoms to the molecular structures. A postprocessing procedure is performed prior to downstream analysis and interpretation of the data. Postprocessing includes data filtering, imputation to account for the missing data and normalization [210]. Data filtering is an important step in order to remove uninformative data while avoiding the loss of biologically meaningful information [217]. Recently, Schiffman *et al.* proposed a data-adaptive pipeline for data filtering procedure [218]. A variety of normalization methods both sample-based and metabolite-based exist. Among these, Variance Stabilization Normalization (VSN), which accounts for sample-to-sample variations and metabolite-to-metabolite variances, has proven to be a suitable and versatile method [219]. However, a recent study recognized 21 different normalization strategies based on the combination of sample-based and metabolite-based methods as consistently well-performing [220]. For an in-depth review of the computational process of the metabolomics studies, we refer the readers to [221].

There are multiple robust tools for each step of the computational workflow (refer to [210, 222] for comprehensive lists of available tools). Metabolomics researchers also enjoy the benefits of existing versatile and comprehensive workflows that cover multiple steps or even the entirety of the metabolomics computational aspects. Examples of highly popular such workflows are XCMS online [223], Galaxy-M [224] and MetaboAnalyst [225]. For a complete step-by-step guide to how to use MetaboAnalyst, we refer the readers to [226]. Moreover, novel approaches and platforms are being rapidly produced. MetaX [227] and JumpM [228] are examples of such novel and potent approaches.

#### Challenges and perspectives

The metabolomics field is rapidly growing with the emergence of innovative technologies such as iKnife [229]. iKnife is able to perform *in situ* MS analysis with applications such as discrimination between normal and malignant tissues with 100% accuracy [230]. Single-cell metabolomics still struggles with challenges such as low throughput and sensitivity as well as computational inefficiencies. Nevertheless, efforts are being made to address such shortcomings [231]. The study of the metabolome is not restricted to the methods discussed in this section. There are also alternative approaches such as isotope tracing fluxomics with the goal of delineation of the distribution of the metabolites in the samples of interest, and matrix-assisted laser desorption ionization-based MS imaging (MALDI-MSI) [232]. Moreover, the diverse advantages of NMR technologies attracted efforts for its synchronization for the current needs of metabolomics studies [233]. These alternative technologies, while providing the research community with improved analytical capacity, bring about their own challenges and inconveniences. Future years are expected to witness increased sensitivity of analytical platforms, improvement of interoperability among computational tools [210], as well as elevated specificity of metabolite biomarkers of cancer and enhancement of pharmacometabolomics (i.e. prediction of drug response through metabolomics) [234].

## Multilayer approaches

**Figure 3:**
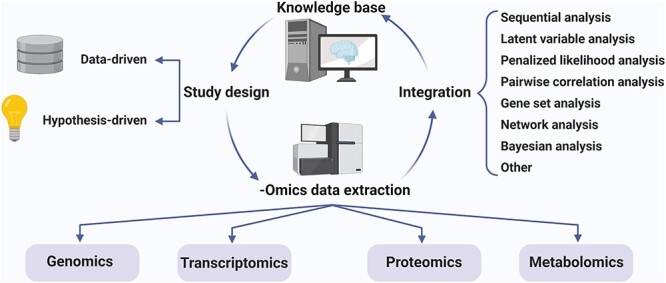
Integrative study of biological phenomena. The first fundamental decision for modern large-scale studies is the choice between hypothesis-driven or data-driven study design. While both types of study designs are applicable, complementary approaches are recommended since hypothesis-driven studies are vulnerable to bias, while data-driven studies are highly prone to false positives [365]. The extracted omics data can be subjected to integration through multiple approaches. The resulting functional data will improve our knowledge base and can serve as a starting point for future studies. Already emerging pipelines demonstrate the clinical utility of the integrative approaches [366]. The integration approaches provided in this figure are based on the categorization in [240]. Sequential analysis: the integration of datasets subsequent to independent analysis. Latent variable analysis: partitioning of samples into functional groups through unsupervised clustering for example by implementation of an expectation–maximization algorithm. Penalized likelihood analysis: outcome prediction through penalized regression. Pairwise correlation analysis: association estimation for related molecule pairs across datasets. Gene set analysis: homogenization of multiple datasets by replacing every molecule with its respective gene and subsequent enrichment of the resulting datasets. Network analysis: using prior knowledge of molecular interactions to provide an environment for integration. Bayesian analysis: utilization of the information in an omics layer as the prior information for the analysis of another through Bayesian approaches.

Although isolated analysis of each of the individual omics layers has substantially contributed to our understanding of a diverse range of biological phenomena, this type of analysis has an inherently limited capacity for characterizing the integrated nature of biological units. When studying the cellular system, its complexity with intertwined and highly convoluted networks of interactions and regulations necessitates a multifaceted approach where different layers of data, generated either through single-layer omics approaches or other means of data acquisition (e.g. studies of molecular interactions, imaging, etc.), are simultaneously analyzed in an integrated manner [235]. Cancer is a systemic disease, and thus, achieving an accurate picture of this perturbation requires homogenization of all the different types of single-layer data through integrative approaches. This is indeed the goal of large-scale efforts such as the Cancer Genome Atlas (TCGA; [13]), which by providing publicly available multilayer data from various tumor types, empower researchers across the globe with an unprecedented capacity for systems-level analysis of cancer (Figure 3).

Integrative approaches have three main advantages. (i) With observations validated across multiple layers of information, they allow for more reliable and representative interpretations; (ii) they can substantially contribute to the delineation of the interplay among molecular levels and shed light on the hierarchy of causation; and (iii) they reduce our blind spots by circumventing our limitations through combined utilization of the technological and computational power in each level.

Notably, omics data are not the only possible source of information that can be purposefully integrated in cancer studies; other types of data such as histopathological information can provide an extended panorama of tumor biology. Reportedly, the integration of histopathological features with molecular data outperforms predictions based on omics data or histopathological information in isolation in various types of cancer [236]. In one such study, an integrative, machine learning-based analysis of histopathological, molecular and clinical data of 538 lung adenocarcinoma patients from TCGA cohorts resulted in an integrated model with more accurate prognostic power for survival outcomes of stage I lung adenocarcinoma patients [237].

The heterogeneity of the generated data across different layers is a major challenge in integrative studies [238]. However, the undeniable advantages of data integration have prompted numerous efforts to overcome its challenges. See [239, 240] for comprehensive explorations of integrative methods, databases and tools. In addition, Supplementary Table S2, see Supplementary Data available online at http://bib.oxfordjournals.org/, describes some of the prominent tools and methods for the integration of multimodal data and their comparative performance. Here, we provide an in-depth description of proteogenomics and network-based data analysis. The former is a remarkable example of how the integration of multiple levels of information can reduce our blind spots and increase the accuracy and reliability of our interpretations and the latter is a major approach for data interpretation and a robust scaffold for data integration and modeling.

### Proteogenomics: vertical integration of genomics, transcriptomics and proteomics data

Since genomic alterations are regarded as the molecular cause of tumorigenesis [7], the emergence of next-generation sequencing (NGS) technologies held the promise to greatly accelerate the identification of pathogenic alterations and thereby facilitate the design of highly effective therapeutic interventions, and indeed, a variety of candidate treatments such as personalized immunotherapy, cancer vaccines and gene therapy are being introduced [241]. However, not all of the patients stratified based on their genomic data benefit equally from the applied therapeutic interventions and the levels of response within each group of patients are diverse [242]. This has been attributed to the fact that most of the currently used treatments target specific proteins rather than genomic alterations and a great number of confounding elements are out of grasp due to the lack of proteomic information [243].

**Figure 4:**
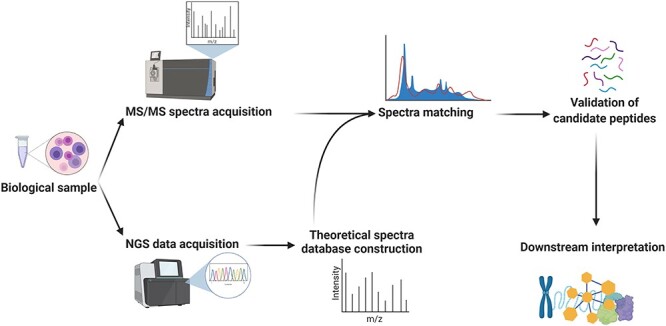
General workflow for the integration of genomics and tandem mass spectrometry data in proteogenomics. The MS/MS spectra of the sample are searched against the theoretical spectra inferred from the NGS data (most commonly RNA-seq) obtained from the same sample. The identified novel peptides should be validated (using PepQuery). The resulting data can be utilized for the study of posttranslational modifications, identification of neoantigens and biomarkers and mutation prioritization in the downstream interpretation. Network-based analysis of these data can provide a critical vantage point for functional study of system perturbations.

Despite recent attempts to predict specific types of PTMs [244], genomics data analysis cannot account for the numerous protein-level adaptation events in the cellular environment [243]. On the other hand, there is a considerable load of somatic mutations in cancer cells that in turn give rise to previously unidentified peptide sequences. Since proteomic analysis relies on previously identified protein sequences (to avoid false peptide sequences in *de novo* sequencing experiments), single-layer analysis of proteomic data is highly limiting in the cancer context. These and other challenges, which will be discussed here, can be addressed through vertical integration of genomics, transcriptomics and proteomics data, which are collectively termed proteogenomics (Figure 4) [245, 246].

#### Experimental workflow and data analysis pipeline

The backbone of proteogenomics studies is the construction of customized protein sequence databases [245]. As previously stated in the proteomics section, the identification of peptides in samples subjected to shotgun proteomics experiments is achieved by matching the spectra against a protein sequence database [247]. However, public protein databases (e.g. UniProt and PDB) do not contain previously unidentified protein sequences such as novel altered proteins that are frequently encountered in tumor-derived samples [248]. To overcome this obstacle, NGS data acquired from the same sample (e.g. via WES, WGS and RNA-seq) can be exploited to construct a customized protein sequence database that contains all the hypothetical protein sequences that can be inferred from the genomics or transcriptomics data and then, match the MS/MS spectra against this sample-specific database [249, 250].

The complexity of the expression system in eukaryotes makes the matching of the proteomics spectra against a customized database predicted from genomics data computationally ineffective and error-prone because the size of such databases will exceed any acceptable threshold [251]. However, customized databases from transcriptomics data are more effective and accurate since they consider only expressed transcripts. To construct a customized protein database from transcriptomics data, raw nucleotide sequences should be assembled into full-length transcripts. There are two approaches for full-length transcript assembly: genome-guided and *de novo* transcriptome assembly. Genome-guided approaches are routinely used for cancer studies. However, coupling these approaches with *de novo* transcriptome assembly approaches is advised [252]. *De novo* transcriptome assembly methods have the advantage of being capable of identifying novel transcripts that can’t be identified through reference-guided methods either due to errors in the reference genome or because they are completely missing (i.e. tumor viruses) [253]. A recent comparative study [252] suggested that the performance of the various existing *de novo* assembly tools is dependent on the study design and the species under study. In the cancer context, where we are usually dealing with human samples, Trinity [254], Trans-ABySS [255], SOAPdenovo-Trans [256] and SPAdes [257] are generally well-performing tools [252]. Merging the results obtained from multiple assembly tools with posterior quality control evaluation is currently considered best practice. Notably, long-read sequencing technologies have the potential to circumvent challenges of *de novo* transcriptome assembly. With PacBio and Nanopore technologies, read lengths of >10 kb are routinely achieved, capturing full-length transcripts.

Multiple tools are available for customized database construction including Galaxy-p [258], QUILTS [249], customProDB [259] and PGA [260]. Importantly, the PGA pipeline is not limited to MS/MS data searching. It incorporates database construction steps that can be done using a genome-guided approach or via a *de novo* transcriptome assembly approach and also includes postprocessing steps including FDR calculation, protein inference and spectrum annotation. In addition, the capacities of Galaxy-p for custom workflow construction prompted the development of comprehensive workflows [261] that encompass the entire computational process of proteogenomics. For a list of available tools and resources for proteogenomics studies, refer to Table 2.

**Table 2:** A list of resources for proteogenomics computational analysis

Tool	Implementation	Description	Reference
*customProDB*	R	Customized database construction from RNA-seq data.	[259]
*FusionPro*	Python & Perl	Identification and annotation of chimeric transcripts.	[379]
*JUMPg*	Perl & R	Customized database construction, database search, filtering and visualization.	[293]
*PepQuery*	Web-based	Validation of novel variants independent of customized database. Also available as a stand-alone tool.	[265]
*PGA*	R	Customized database construction and novel peptide identification.	[260]
*PGTools*	Perl & Python	Customized database construction, FDR estimation, protein identification and annotation, visualization.	[380]
*ProGeo-neo*	Python	Neoantigen identification, classification and prioritization.	[381]
*PROTEOFORMER 2.0*	Python & Perl	Proteoform identification through proteogenomic analysis of ribosome profiling and MS/MS data.	[382]
*QUILTS*	Web-based	Customized database construction.	[249]
*SAAVpedia*	Web-based & Python	User-friendly single amino acid variant prioritization.	[383]
*Spritz*	Windows	User-friendly customized database construction. Importantly, it accepts raw RNA-seq data as input and automatically performs preprocessing through utilization of 23 tools.	[384]

The process of matching MS/MS spectra against a customized database is achieved by utilizing database search engines such as X!Tandem, MS-GF+ [262] and Comet [263]. Among these, the widely used X!Tandem software has been shown to have the highest false negative rate, and hence, it is not recommended to exclusively use this engine [264]. Since effective quality control methods for novel peptide identification can be utilized downstream of the matching process, a high level of false positive can be tolerated. Hence, the best approach in this step is to combine the results of multiple search engines to gain a more comprehensive collection of putative novel peptides. Novel peptides that have been identified through the matching step can then be further validated. PepQuery [265] is a freely available tool that can be applied as an optional quality control step and can significantly reduce false positives. The definitive validation of identified novel peptides, however, can be achieved through targeted proteomics assays [243].

#### Applications

There is a variety of molecular events that can potentially give rise to a wide range of protein alterations such as chimeric proteins or single amino-acid variants in cancerous cells. However, not all of these events result in expressed proteins and even if expressed, the resulting proteins might be unstable and subjects to early degradation. Proteogenomics is an ideal approach for protein-level validation of the stable expression of these molecular events [246]. Moreover, protein-level analysis of current gene models and their somatic variations by means of proteogenomics enables the validation or correction of previous predictions of the sequence, structure and ultimately the function of the respective proteins [246, 266]. Additionally, deregulation of alternative splicing in cancer under the influence of perturbed splicing factors and altered signaling cascades is a known phenomenon [267, 268]. Alternatively spliced isoforms can not only serve as tumor-specific biomarkers but can also provide stage-specific signatures and putative therapeutic targets [80]. Empowered with the capacities of both transcriptomics and proteomics, proteogenomics proves to be a competent approach for studying oncogenic splice variants and specific pipelines toward this purpose have already been developed [269].

PTMs are known to play essential roles in the biology of cancer cells [143, 144]. Genomic alterations in cancer can have profound effects on protein modifications (e.g. through the addition or disruption of modification sites or alteration of PTM regulator proteins) and in turn on the signaling cascades and regulatory networks of cancer cells [251, 270]. Since PTMs cannot be accurately predicted from genomics data, proteogenomics can become the tool of choice for exploring the effects of aberrations in the genome on the downstream PTM alterations [271]. In addition, it is now widely accepted that quantitative mRNA expression data are not an ideal indicator of protein expression levels and the extent to which they biologically correlate is a matter of debate [272]. Since protein expression levels are of importance both for functional inferences and therapeutic interventions, accurate measurement of protein expression levels is crucial [243]. Proteogenomics studies can not only provide us with protein expression data, but they also have the potential to deepen our understanding of the biology of this difference in expression levels.

The host immune system is known to be effective in the elimination of cancer cells [273]. For the host immune system to be able to confront cancer cells, neoantigens, which are predominantly results of the processing of altered proteins by the antigen processing pathways, should be presented as human leukocyte antigen (HLA) ligands at the cell surface and be identified by T-cell surveillance [268, 274]. The process of immune response to cancer cells is being studied with the goal of designing therapeutic interventions known as cancer vaccinations that attempt to elicit the T-cell immune response against cancer cells [275–277]. Proteogenomics can greatly accelerate the pace of neoantigen discovery and by providing candidate clonal neoantigens result in a more efficient vaccination process [278, 279]. Moreover, proteogenomics studies can help delineate the underlying mechanisms of immune system evasion by cancer cells [280].

The above-mentioned applications can be used to filter more important genomic alterations, distinguish between driver and passenger mutations [281] and make for more efficient biomarker discovery [282–284]. A recent study [266] showcased the massive potential of proteogenomics studies from unraveling uncharted aspects of cancer biology to opening new avenues toward precision oncology. From PTM analysis of proteins to prioritization of somatic copy-number alterations, they exploited the full potential of current proteogenomics technologies. Importantly, they demonstrated that proteogenomics studies can result in more efficient unified multiomics cancer subtypes that can serve to acquire an enhanced ability for prognosis, diagnosis and precision interventions.

#### Challenges and perspectives

A long-standing challenge in the field of proteogenomics is the appropriate FDR estimation for matched peptides after database search [246]. As discussed in the proteomics section, a widely used approach is the target-decoy search strategy [163]. Since assuming the same FDR for both novel and previously identified peptide sequences is an underestimation of the FDR value for novel peptides, the efficacy of this method in proteogenomics studies has been questioned and substitute approaches such as separate FDR estimations for novel and previously identified peptides have been suggested by Nesvizhskii *et al.* [246]. Wen *et al.* [264], however, in a comparative study of FDR estimation methods utilized the prediction of retention time for peptides in comparison with the actual observed values as an evaluation metric for different quality control strategies and identified global FDR estimation by target-decoy search (in order to attain a high level of sensitivity) with a posterior filtering step to restrict false positives (using PepQuery) as the best approach for neoantigen discovery.

Although targeted MS-based assays hold great promise for the clinical translation of the discovered biomarkers through proteogenomics studies, there are still challenges that should be addressed [243]. Targeted multiple reaction monitoring assays can be used not only to validate the results of proteogenomics analyses but can also provide clinicians with a cost-effective multiplexed platform that can analyze a high number of target proteins from a variety of sample types (e.g. urine, secretions, etc.) with satisfying sensitivity and specificity. However, there is still room for improvement since the sensitivity is not enough for dilute samples and single-cell analysis [285].

Recent advancements in proteomics technologies [286, 287] and clinically valuable demonstrations such as the possibility of a microscaled proteogenomics study of tissues as small as 25 μg [288] are setting the stage for the emergence of a more precise and cost-/time-effective landscape for proteogenomics. Moreover, single-cell proteogenomics is evolving and has the potential to considerably increase our understanding of intratumoral heterogeneity [289–291]. It is expected that a greater number of researchers will join this field in the years to come. However, the high number of existing tools that provide complementary results and should be utilized in combination with one another in multiple steps of the study [264, 284, 292] is probably a prohibitive element in attracting new researchers to the field. Other prohibitive elements are the required computational expertise and the lack of unified and comprehensive databases with user-friendly interfaces that are specifically tuned for proteogenomics studies. Although efforts have been made to provide comprehensive workflows for different study goals [293, 294], international collaborations are required to overcome existing challenges and provide gold standard workflows for proteogenomics studies.

### Network-based data integration

A huge amount of information regarding the interactions among molecules and biological pathways is stored in public data repositories such as STRING [295], BioGRID [296], InnateDB [297], KEGG [298], Reactome [299], VMH [300], WikiPathways [301], etc. These data are generated either from *in vivo* and *in vitro* experiments or from *in silico* predictions [302] and are essential in providing a system-based context for omics data. Biological systems in the form of interaction networks and pathways can serve as frameworks on which omics-driven data can be integrated, analyzed and interpreted [303, 304].

Combining the prior knowledge of interactions in the form of networks and pathways with genome-wide data generated through single-layer omics approaches is used to overcome issues in the interpretation of omics data by providing a larger context. On the one hand, omics data on their own are merely a representation of existing molecules and their abundances at a particular point in time. Extracting patterns and understanding the underlying mechanisms of a condition from an omics dataset in isolation is challenging [305]. On the other hand, molecular interaction networks and pathways, although highly informative, do not account for the dynamics of the cell in different states and phases. The integration of interaction networks and pathways with omics datasets facilitates pattern detection and allows the study of the dynamic nature of the cell [306]. This is of particular importance for understanding the mechanisms of complex multistage diseases such as cancer. This integrative approach has been shown to be superior to the isolated analysis of either networks or omics data [307].

**Figure 5:**
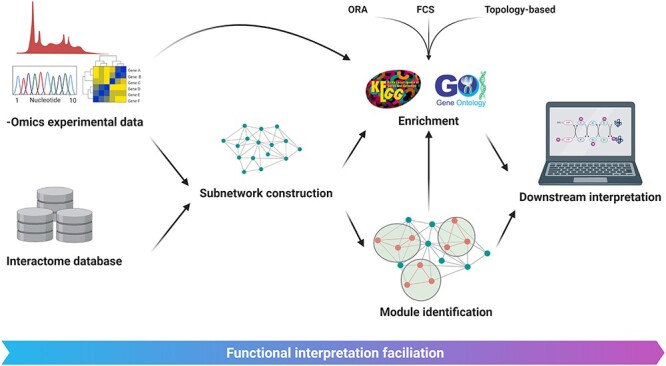
General workflow for the network-based analysis of omics data. The constructed subnetworks from the integration of the omics-driven data and prior knowledge of molecular interactions can be subjected to module identification or enrichment analysis. The identified modules can also be enriched to yield functional information. Note that it is possible to enrich the omics data independent of the subnetwork construction process. An example of downstream interpretation is to demonstrate multiomics data in multilayered networks for computational and/or visual pattern detection. Going from either raw omics data or interactome databases to subnetwork modules and enriched data, the complexity decreases, and the data are constantly narrowed down to yield functional information. ORA, overrepresentation analysis; FCS, functional class scoring.

An important advantage of this integrative approach is the provided capacity for topological analysis of the identified significant molecules (e.g. downstream/upstream position in a given pathway, centrality parameters [308], etc.). It is widely accepted that the upstream position of a molecule in a pathway can be considered as a predictive measure for biological significance [309]. In addition, the centrality of a node in a given network, measured by various parameters (e.g. degree, betweenness, etc.), is a validated implication for distinct importance. Indeed, aberrations in central nodes have been shown to play vital roles in tumor development [310]. Thus, alterations in structure or function (e.g. differential expression/abundance) of a given molecule under certain conditions combined with its topological features can help prioritize candidate molecules (e.g. possible driver molecules) for further studies [306]. Identification of patterns that are unlikely to occur randomly is another important theme in network biology. These patterns include motifs and modules. Motifs are recurring small subgraphs whose interactions form the overall behavior of the complex network. Alterations in these motifs are central to cancer biology and the search for core motifs in cancer-related pathways is valuable for biomarker, therapeutic target and subtype discovery [311]. Modules are larger subgraphs that are highly connected internally and are involved in specific processes. Modules are extensively investigated for the identification of cancer driver pathways and genes and are explained in more detail in further sections.

Guilt-by-association is another concept widely used for biological inference of topological properties of molecular networks in cancer biology [312]. This notion posits that molecules in topological proximity of each other are potentially functionally related. This is utilized in multiple ways in cancer investigations. For example, proteins of unknown significance in close topological proximity of known drivers of cancer can be investigated as candidate infrequently mutated proteins of functional importance in cancer. Alternatively, proximity as a proxy for overlap in function can be exploited to avoid utilization of redundant molecules for survival analysis, leading to higher efficacy of prognostic biomarkers [312].

Biomarkers and gene signatures identified from network-based approaches have been shown to be more reproducible [313]. In addition, network-based approaches allow the study of perturbations in specific interactions among molecules (e.g. allosteric regulations, posttranslational processing, etc.) [307, 314]. Deviation of these interactions from normal status is an essential factor in tumorigenesis and cancer progression [141]. Collectively, network-based analysis of cancer has been successfully implemented in cancer driver pathway identification, driver gene discovery, somatic mutation prioritization, biomarker and therapeutic target discovery, cancer subtyping and patient stratification [312].

The first step of the network-based analysis of omics data is to construct a context-specific subnetwork from generic data repositories of molecular interactions and pathways [311, 315]. These subnetworks represent the parts of the system that are being studied and are constructed based on the experimentally acquired omics datasets. Depending on the goal of the study, different types of networks can be constructed including gene–gene and gene–protein interaction networks, signaling pathways or a combination of these for a more comprehensive analysis. The most widely used networks are PPI networks [316] and genome-scale metabolic models [317]. The constructed subnetworks can then be amended with the results of a pathway enrichment analysis or can be mined for active module identification (Figure 5). These steps along with the visualization approaches are discussed in more detail below.

#### Subnetwork construction

Generic databases of biological interactions and pathways are still far from complete [318, 319]. However, the goal of these repositories is to capture the entire repertoire of molecular and/or cellular interactions. Meanwhile, depending on the significant molecules identified in the omics dataset under analysis, only a minor subset of these interactions is relevant. Hence, the first step for network-based analysis of datasets is to construct a context-specific subnetwork. In addition to the significant molecules, identified via omics data analysis, subnetworks commonly incorporate all the known molecules that are in direct interaction with them [315]. These extra nodes provide new perspectives for a more comprehensive and accurate network interpretation.

Network-based approaches can greatly facilitate multiomics data integration and analysis [303]. Multiple levels of omics data produced from different single-layer techniques can be layered upon a single network to achieve a more holistic view of the perturbed system [307]. Alternatively, it is possible to construct multiple networks from different levels of omics data. The comparison of these networks can provide a deeper and more accurate view of the system under investigation and result in more reliable conclusions [320]. Several algorithms such as AMARETTO [321] and iOmicsPASS [322] facilitate network-based data integration. Interestingly, AMARETTO is able to integrate phenotypic information such as radiography data with multiomics data. This practical approach has been shown to be effective in identifying candidate cancer driver genes [314].

#### Module identification

The constructed subnetworks are usually very complex and often referred to as hairballs. While almost impossible to manually identify functional patterns in these subnetworks, graph mining algorithms can be applied to identify functional units of the large subnetwork known as modules [323]. Modules can be regarded as sets of densely connected nodes with an overall limited connection to the rest of the network [324, 325]. An important property of biological systems is that molecules with similar functions closely interact with one another and tend to cluster together in biological networks [325]. Hence, each module can be assigned a specific biological function. If a subnetwork is constructed based on differential expression/abundance of molecules under a certain condition, modules in this subnetwork are expected to represent perturbed parts of the system that gave rise to the condition under investigation.

Since the disruption of certain pathways (e.g. apoptosis, proliferation, etc.) is common to almost all cancer types [141], it is logical to consider that genes that harbor driver mutations should at least to some extent cluster together in modules [326, 327]. It is expected that modules containing genes that are known to be involved in tumorigenesis and cancer progression can be utilized to predict novel cancer driver genes. Moreover, module identification can facilitate the identification of co-occurring cancer driver mutations [328].

The analysis of network modules facilitates the discovery of common disease mechanisms, disease subtypes or the mechanics of response to drugs [329]. Interestingly, biological networks are often hierarchically organized, where for example a group of small, interconnected modules can be clustered together to form larger modules. Researchers can use these hierarchies to adjust the magnification of the analysis for a more biologically relevant interpretation [330]. Methods such as hierarchical Hotnet are specifically developed for cancer studies to identify these module hierarchies and predict cancer driver genes [331].

Commonly used methods for module identification [332–334] first score nodes and edges based on criteria such as differential expression and experimentally validated PPIs, respectively. Then, a scoring system based on the aggregated scores of all the members of a hypothetical module is formulated. An algorithm is used to identify optimal modules (those with the highest scores). In the final step, the identified modules are queried for their statistical significance in relation to the investigated hypothesis [329].

Multiple classes of algorithms have been implemented for module identification, including diffusion-based algorithms and algorithms based on the prize-collecting Steiner tree problem [312]. Briefly, diffusion algorithms consider significant molecules as sources of a phenomenon such as heat diffusion that spreads through the edges of the network until equilibrium is achieved. Here, the goal is to find regions of the network with the most influence over them (i.e. hot regions) as these regions represent highly active modules. Prize-collecting Steiner tree algorithms seek to find modules optimized to contain the highest number of prizes (significant nodes) while minimizing the number of edges. Some algorithms [335] also exploit specific properties of tumors such as mutual exclusivity (i.e. activation/inactivation of a second driver molecule functionally related to an already perturbed molecule is obsolete and rarely observed in a single tumor).

jActiveModules [332] is a widely used plug-in for network visualization software Cytoscape [336]. It can be used for module identification and can determine whether modules are common in multiple states. jActiveModules scores all the nodes in a network based on the *P*-values from a differential gene expression analysis and has a scoring function to determine the statistical significance of any given module. First, it assigns an active or inactive state to each node in a subnetwork (with a 0.5 probability). Then, for a defined number of iterations, it selects a random node, toggles its state (active/inactive) and recomputes the module’s score. If the aggregated score of the module has increased, it keeps the node in its new state. Otherwise, it keeps or changes its state with a defined probability. The process continues until a local optimum is achieved. The identified module might not be the module with the global maximum score, but regardless it is of biological interest.

Approaches for module identification are not limited to what has just been described. For example, in [337], the authors proposed a novel module-identification pipeline. In this method, gene–gene correlation networks are constructed from omics data from two conditions under comparison. Then, the networks are separately integrated with *a priori* knowledge of interactions to identify modules. Thereafter, enriched modules (e.g. those significantly associated with upregulated genes in a certain condition) can be identified and potentially be used for predictive or diagnostic purposes. A few outstanding challenges regarding the existing methods and the overall approach should be considered. There is a lack of a strong correlation between mRNA and protein abundance [338]. As a consequence, utilizing the mRNA profile on its own as the source for subnetwork construction would result in an inaccurate representation of the actual system. iOmicsPASS [322] is a recently developed algorithm that takes this issue into account by integrating transcriptomics and proteomics data. iOmicsPass predicts phenotypic groups based on the joint expression pattern of the nodes within densely connected modules. The algorithm has been shown to be effective for predictive module identification especially when dealing with smaller datasets. Another major problem is that it is possible for a single molecule to be shared among multiple biological modules. Current methods, however, are not computationally effective in identifying overlapping modules [327]. Furthermore, despite a considerable rate of development of novel methods, there is a lack of standard benchmarks for validation and comparison of suggested methods [329]. In addition, it should be noted that the assumption that disease-related molecules cluster together in interaction networks does not always hold for a complex condition such as cancer [327].

#### Pathway enrichment analysis

Pathway enrichment analysis is a common approach for identifying disrupted molecular processes and pathways underlying a certain condition [339]. The central idea is to identify common pathways that a set of molecules (e.g. differentially expressed genes) is associated with. This reduces the contextual complexity of the system and simplifies the interpretation of omics datasets by taking advantage of prior knowledge about biological processes [340].

Three generations of pathway enrichment methods have been developed [340]. The first generation was termed overrepresentation analysis (ORA). In this generation of methods, a list of significantly differentially expressed molecules, based on *P*-value and/or fold change filters, is compared against previously compiled functional lists of molecular processes to identify overrepresented pathways. DAVID [341] and WebGestalt [342] are among the widely used tools that exploit ORA algorithms. A major drawback of ORA is that by defining filters, we risk the omission of important molecules [343]. Moreover, ORA algorithms treat all the molecules that passed the defined filters as equally significant [304].

The second generation of pathway enrichment methods is known as functional class scoring (FCS). Instead of using predefined filters, FCS algorithms require an input list of all the evaluated molecules, along with values corresponding to their level of differential expression (e.g. fold-change or *P*-value) [340]. In these methods, all the input molecules are statistically ranked and the overrepresentation of pathways is analyzed with the impact of each molecule in consideration [304]. A pitfall in this approach is that the analysis can become biased toward a few molecules that have been identified as very significant. Gene set enrichment analysis (GSEA) [344] is a widely used algorithm belonging to the second generation of pathway enrichment analysis methods. GeneTrail [345] is a popular and freely accessible web service that provides users with both ORA and FCS algorithms for pathway enrichment analysis.

The most recent generation of pathway enrichment methods was developed with the goal to maximize the utilization of prior biological knowledge [346]. This generation of pathway enrichment algorithms incorporates the topological features of nodes in biological networks (e.g. upstream or downstream position in the pathway, degree and betweenness) as additional weighting factors in the enrichment process [309, 315]. In addition, in topology-based methods, the analysis is not limited to input molecules but other molecules with close connections to input molecules can be incorporated to identify relevant pathways. Studies indicate that topology-based methods outperform conventional methods (ORA and FCS) both in genomics and metabolomics enrichment analyses [324, 347]. This generation of algorithms provides better capacity for the analysis of molecular interactions and understanding the underlying mechanisms of a condition. In general, there is no single best-performing tool for topology-based enrichment analysis. However, a recent comparative study [324] identified DEGraph [348] as the superior method among the nine algorithms investigated.

Overall, some major challenges remain for pathway enrichment analysis. In a recent study [347], Nguyen and co-authors found that all of the tested pathway enrichment methods with the exception of GSEA are prone to report false positives. GSEA, on the other hand, suffers from low sensitivity. Furthermore, the Fisher’s exact test, while a highly utilized method, performed poorly in this study and produced a significant number of false positive results. Hence, highly popular platforms such as DAVID, which use this method, should be treated with extra care.

Most comparative studies focus on gene expression data and the results of these studies are not necessarily applicable to other data types (for a list of methods and tools utilized in enrichment analyses and their comparative performance derived from comparative studies, refer to Supplementary Table S3, see Supplementary Data available online at http://bib.oxfordjournals.org/). Considering the importance of other layers of information in cancer studies, this should be considered in future developments. One tool that already supports other layers of information, including genomics, transcriptomics, proteomics, miRNAomics, epigenomics, etc., is GeneTrail [345]. In addition, although studies indicate the superiority of topology-based enrichment methods, it is still not sufficiently recognized. It would be ideal if popular and user-friendly portals of enrichment analysis would incorporate topology-based approaches in order to make these methods accessible to a wider range of researchers.

The current lack of gold standard methods for pathway enrichment analysis coupled with the plethora of existing approaches makes the selection of a suitable method a challenging task. This is especially burdensome for researchers with limited computational expertise. With that being said, there are a number of user-friendly web-based platforms such as MetaboAnalyst [225] and Metascape [349] that offer users a comprehensive pipeline for pathway enrichment analysis. Metascape (https://metascape.org/) takes advantage of multiple databases as its resource for systems-level analysis of datasets. It provides powerful computational abilities with a simplified and user-friendly interface designed for researchers with minimal computational expertise. Since outdated data can severely impact the quality of analysis results [350], an important feature of Metascape is the monthly data synchronization with the updated information in data repositories. The workflow of Metascape can also be modified by users with more advanced computational skills to meet the requirements of individual studies. Moreover, it can be utilized for cross-omics comparisons of multiple gene lists and integrated analyses. Similar to DAVID, the resulting enriched terms in Metascape are clustered and nonredundant. The results can also be exported to Cytoscape for further analysis.

#### Network visualization

Through visualization, large amounts of data can be made more accessible for convenient pattern detection and interpretation [351]. Whether it is in the form of processed networks or categorized and functional tables, the goal of the visualization process is to reduce the overwhelming complexity of large datasets and make them more readily interpretable. Many tools such as Cytoscape [336], PaintOmics [352] and Omicsnet [353] are developed with the objective of simplifying the visualization process and offering users a wide array of options to modify how their data are represented.

Cytoscape is a widely used freely accessible platform that provides users with an interactive interface and powerful tools for network visualization and analysis. Cytoscape’s feature set can be expanded by adding plug-ins developed by the community for specific computational tasks. Omicsnet [353] is a recently developed web-based visualization tool (www.omicsnet.ca/) that provides users with a 3D structure for visualization and analysis of large networks. It can incorporate multiple heterogeneous datasets in a single subnetwork. Moreover, by taking advantage of various structural layouts such as spherical and multilayer layouts, it facilitates network analysis and reduces the overwhelming complexity of large networks. In addition, it provides users with a variety of functional and topological analysis tools including module identification and pathway enrichment analysis.

#### Challenges and perspectives

Although there are numerous methods and tools developed to tackle the variety of problems associated with the network-based analysis of omics data, this approach to data analysis is still in its infancy. Whether it is a matter of reliability of the analysis or a matter of providing equilibrium between the amount of lost data and precision, a number of challenges remain for the community to address.

The quality of network analysis results can only be as good as the quality of the input data. Besides the quality of omics data, a major challenge in this field is incomplete or inaccurate information in network and pathway databases that has been shown to greatly affect the analysis process [350]. Hence, efforts to validate and expand the information in these databases are of essential importance. In addition, analysis tools need to regularly update their knowledge base to keep up with the expansion pace of the source databases. Moreover, limited overlap among interactome databases means that they should be used in combination for more comprehensive results [347].

A simple widespread approach for subnetwork construction is the inference of relevant nodes based on significantly differentially expressed/abundant mRNAs or proteins. However, two caveats should be considered when opting for such approaches. First, since there is evidence against a strong correlation between mRNA and protein levels [272], the accuracy of utilizing mRNA expression levels for subnetwork construction is questionable. Second, phenomena such as somatic mutations, PTMs and alterations in cellular localization can functionally affect PPIs. These alterations might be overlooked when PPI subnetworks are constructed solely based on mRNA expression or protein abundance. When this is coupled with inaccuracies and incompleteness of current PPI databases, it becomes clear that constructed subnetworks based on differential mRNA expression or protein abundance do not necessarily provide accurate representations of the altered cellular interaction networks. Integrative approaches can ameliorate this flaw to a great extent. For instance, using integrative analysis approaches prior to subnetwork construction, one can establish a list of candidate significant molecules (e.g. genes with both somatic mutation and differential expression, overexpressed genes with hypomethylation, etc.) and subsequently create a subnetwork by mapping these molecules to the human interactome [354]. Alternatives include more sophisticated methods where a list of candidate molecules is not determined *a priori*. For example, in the very recently introduced EMOGI method specifically developed for cancer data exploration [355], novel candidate cancer genes are predicted through a machine-learning approach that uses a generic PPI network with a multiomics feature vector for each node along with lists of high-confidence cancer/noncancer genes as input. However, only a limited number of user-friendly tools allow for a network-based multiomics data analysis. Moreover, current tools that provide the capacity for this type of analysis are not comprehensive with regards to the types of integration they can carry out.

Recently, efforts have been made to systematically compare the plethora of existing methods. These studies analyzed current popular methods from different perspectives, deducing different existing challenges in the field, from the lack of a uniform distribution of *P*-values under the null condition for enrichment analyses to the absence of a perfect method for all the study goals [324, 347].

An exciting future awaits the network-biology approaches. Single-cell multiomics technologies provide a highly potent data source for the construction of multilayered networks providing holistic views of individual cellular systems. Moreover, it opens a great opportunity for understanding intratumoral heterogeneity [356]. From the enhanced capability to unravel the complex underlying mechanisms of cancer to drug repurposing [357] and precision medicine [358], network-based approaches facilitate the translation of raw biological data of single-layer omics experiments to practical knowledge and possible interventions.

### Successful implementations of integrative approaches in cancer research

With significant growth during the last decade, high-throughput technologies prompted many studies with results of clinical relevance. The search for molecular markers predictive of the response to specific types of treatment is a hot topic in precision oncology and many studies provide encouraging results. For instance, in a study by Taber *et al.* [359], sequential analysis of genomics, transcriptomics and proteomics data resulted in the identification of a subgroup of muscle-invasive bladder cancer patients with high genomic instability and nonbasal/squamous expression subtype that were highly responsive to cisplatin-based chemotherapy, while patients with low genomic instability and basal/squamous expression subtype showed poor response. In another study, proteogenomics analysis of HPV-negative head and neck squamous cell carcinoma shed light upon multiple clinically significant aspects of this malignancy [360]. In addition to providing insights into the underlying biology of this type of cancer, they identified multiple potentially druggable targets. Interestingly, this study proposed that amplification of EGFR does not necessarily correlate with the prevalence of EGFR ligands, suggesting that the investigation of EGFR ligand abundance is a more appropriate strategy for prediction of response to treatments with anti-EGFR monoclonal antibodies.

The interplay between molecules is best explored through network analysis. In a remarkable pan-cancer network-based integration of genomics and transcriptomics data of 9738 samples from 20 TCGA cohorts, Paull et al. [361] identified 407 master regulator (MR) proteins responsible for channeling the functional effects of the plethora of genomic aberrations to specific gene expression signatures across tumor types. These proteins were categorized into 24 MR modules, each involved in the regulation of specific hallmarks of cancer. They proposed that based on the status of these 24 modules (activated/inactivated) in each individual, patient-tailored combinations of drugs that specifically target these modules can be administered with precision.

In addition, although in its infancy, single-cell multiomics is an emerging mighty technology. Perhaps, the most profound contribution of single-cell technologies is that they allow us to dissect intratumoral heterogeneity at individual cell resolution and explore common cancer type- or subtype-specific patterns of heterogeneity among cellular clusters. The delineation of these patterns can enhance our understanding of how tumors with specific origins exhibit certain properties (e.g. metastasis, drug resistance, etc.), yielding insights into their assailable aspects and providing new means for patient stratification [131]. Single-cell multiomics has the capacity to uncover intratumoral heterogeneity across layers of molecular information and provide us with a systems-level understanding of this phenomenon. Indeed, an integrative study of mRNA and protein levels at single-cell resolution evaluating the effect of BMP4 (a proposed therapeutic agent for glioblastoma [362]) on early-passage glioblastoma cultures [363] identified extensive heterogeneity in how subpopulations of cells respond to BMP4 treatment. Utilizing the mRNA and protein information in complement, they concluded that while all of the treated cells activated the BMP4 pathway, a subset of cells escapes proliferation suppressive effects of BMP4 treatment through a TNC protein-dependent mechanism. Together, such studies illustrate the massive potential of integrative approaches in deepening our understanding of tumor biology and directing clinical efforts toward precise patient stratification and treatment.

## Conclusion

Current omics technologies and computational advancements provide unprecedented capacity to study cancer etiology and underlying mechanisms, discover clinically applicable diagnostic and predictive biomarkers, identify therapeutic targets and develop therapeutic interventions. Despite significant progress in the field, various uncharted territories remain to be explored. The fact that no driver mutation could be identified for 5% of the cancers [16] or the unknown exact basis for metastasis [66] highlights the existence of fundamental gaps in our knowledge. Until these fundamental shortcomings in our knowledge persist, our inability to design highly effective therapeutic interventions is not surprising. With the enhancement of our knowledge during the last decades, it is becoming evident that cancer should no longer be viewed as a disease of the genome but should rather be regarded as a disease of the cellular system. Rapid advances in technologies and methodologies are paving the road for more effective study of cellular systems and their perturbations. However, the dispersion of the plethora of bioinformatics tools, the lack of benchmarked gold standard methods and the required computational skills are major prohibitive elements. There is an ever-growing need for user-friendly workflows that have been adjusted for specific study goals. The extension of current comprehensive platforms such as Galaxy [364] that allow for designing and utilizing readymade workflows for a very wide range of omics experiments will result in further facilitation of data analysis processes.

**Key Points**

- Systemic perception of cancer is essential for the design of effective interventions.
- High-throughput technologies are the main arteries of systemic studies of cancer.
- Emerging data integration approaches are rapidly altering current paradigms of oncology.
- Vertical integration of omics data is capable of addressing multifaceted challenges.
- Network-based data analysis is a major asset in data integration and interpretation.

## Supplementary Material

Supplementary_Table_S1_bbab343

Supplementary_Table_S2_bbab343

Supplementary_Table_S3_bbab343

## Abbreviations

1H NMR: Proton nuclear magnetic resonance; CASAVA: Consensus Assessment of Sequence And Variation; cfDNA; cell-free DNA; CHIP: Clonal hematopoiesis of indeterminate potential; Co-IP: Co-immunoprecipitation; ctDNA: circulating tumor DNA; DGE: Differential gene expression; ESI: Electrospray ionization; FCS: Functional class scoring; FDR: False discovery rate; GATK: Genome Analysis Toolkit; GC–MS: Gas chromatography–mass spectrometry; GSEA: Gene set enrichment analysis; HLA: Human leukocyte antigen; HMDB: Human Metabolome Database; HPPP: Human Plasma Proteome Project; LC–MS/MS: Liquid chromatography–tandem mass spectrometry; MALDI-MSI: Matrix-assisted laser desorption ionization-based mass spectrometry imaging; MR: Master regulator; NGS: Next-generation sequencing; ORA: Overrepresentation analysis; PCAWG: Pan-Cancer Analysis of Whole-Genomes; PPI: Protein–protein interaction; PRM: Parallel reaction monitoring; PTM: Post-translational modification; SRM: Selected reaction monitoring; TAP: Tandem affinity purification; TCGA: The Cancer Genome Atlas; TMT: Tandem mass tag; UMI: Unique molecular identifiers; VAF: Variant allele frequency; VSN: Variance Stabilization Normalization; WES: Whole-exome sequencing; WGS: Whole-genome sequencing; XL-MS: Crosslinking-mass spectrometry; lncRNA: long noncoding RNA; miRNA: microRNA; ncRNA: noncoding RNA; sncRNA: small noncoding RNA.

## Notes

**Mohammad Reza Karimi**, graduated in molecular and cell biology from the Department of Cell & Molecular Biology, Semnan University, Semnan, Iran. Experienced in genome-wide data handling and biological network analysis.

**Amir Hossein Karimi**, graduated in molecular and cell biology from the Department of Cell & Molecular Biology, Semnan University, Semnan, Iran. Experienced in Omics data analysis and cancer systems medicine.

**Shamsozoha Abolmaali**, is Assistant Professor in the Department of Cell & Molecular Biology, Semnan University, Semnan, Iran. Expert in genetic engineering, biotechnology and experiment design.

**Mehdi Sadeghi**, is Assistant Professor in the Department of Cell & Molecular Biology, Semnan University, Semnan, Iran. He is interested in computational systems biology, Omics data integration and cancer systems medicine.

**Ulf Schmitz**, is Associate Professor of Bioinformatics in the Department of Molecular & Cell Biology, James Cook University, Townsville, QLD 4811, Australia. He is interested in computational RNA biology and systems medicine and develops integrative workflows for the analysis of gene regulation in cancer.
