# Supplementary material for: Prospects and challenges of cancer systems medicine: from genes to disease networks
Source: Brief Bioinform. 2021 Sep 1;23(1):bbab343. doi: 10.1093/bib/bbab343 (PMC8769701; doi:10.1093/bib/bbab343)
Supplement: Supplementary_Table_S1_bbab343 [file supplementary_table_s1_bbab343.pdf]

Table S1. A list of relevant researches and their contributions to the field of systems oncology

Reference	Category	Tumor/sample type	Experiment	Type of analysis	Outcome
Panou et al. 2018	Genomics	Peripheral blood or saliva and fresh-frozen paraffin-embedded specimens of malignant mesothelioma patients	Targeted Sequencing	Germline and somatic variant analysis	12% of malignant mesothelioma patients had defects in cancer susceptibility genes. Identification of HR deficiency in a considerable portion of patients provides rational for PARPi treatment
Alexandrov et al. 2013	Genomics	Pan-cancer	WGS, WES	SV analysis	Identification of shared and distinct mutational signatures
Alexandrov et al. 2020	Genomics	Pan-cancer	WGS, WES	SV analysis	Pan-cancer exploration of mutational signatures and their associations with exogenous and endogenous exposures and defects in DNA maintenance
Itamochi et al. 2017	Genomics	Ovarian clear cell carcinoma	WGS	Germline and SV analysis, CNV analysis	Identification of potential prognostic biomarkers and therapeutic targets
Bartoschek et al. 2018	Transcriptomics	Cancer-associated fibroblasts of genetically engineered mouse model of breast cancer	Single-cell RNA-Seq	Gene expression analysis	Identification of three distinct transcriptomic subpopulations of cancer-associated fibroblasts and potential prognostic biomarkers or markers of drug response
Patel et al. 2014	Transcriptomics	Primary glioblastoma	RNA-Seq, Single-cell RNA-Seq	Gene expression analysis, CNV analysis, Clustering,	Contribution to delineation of intra-tumoral heterogeneity landscape of glioblastoma
Zhu et al. 2020	Transcriptomics	Colorectal adenoma/Colorectal cancer	RNA-Seq	Differential expression analysis	Potential predictive biomarker and therapeutic marker discovery
Eswaran et al. 2013	Transcriptomics	Breast cancer	RNA-Seq	Alternative splicing analysis	Exploration of subtype specific splicing events and identification of common splicing events in breast cancer
Yamada et al. 2018	Transcriptomics	Colorectal cancer	RNA-Seq	Differential expression analysis	Exploration of long non-coding RNA landscape in colorectal cancer and identification of potential biomarkers and therapeutic targets
Bouchal et al. 2015; Procházková, Lenčo, and Bouchal 2017	Proteomics	Breast cancer	LC-MS/MS, SRM	Differential protein abundance analysis	Identification and validation of prognostic biomarkers
Swiatly et al. 2018	Proteomics	Benign ovarian tumor and ovarian cancer	LC-MS/MS	Differential protein abundance analysis	Identification of markers for screening and detection of ovarian cancer
Guerreiro et al. 2020	Proteomics	Oral squamous cell carcinoma cell line, Pancreatic ductal adenocarcinoma cell line, Melanoma brain metastasis cell line	LC-MS	Protein content analysis and comparison for extracellular vesicles	Identification of potential cancer-specific diagnostic biomarkers
Zhou et al. 2020	Proteomics	Plasma samples of gastric cancer	LC-MS/MS	Differential protein abundance analysis	Potential diagnostic biomarker identification
Luo et al. 2020	Metabolomics	Pancreatic cancer	Precision-targeted LC-MS	Differential metabolite abundance analysis	Identification of five plasma metabolite biomarkers that can effectively diagnose pancreatic cancer. Identification of two biomarkers that can potentially diagnose progression and metastasis of pancreatic cancer
Bruzzone et al. 2020	Metabolomics	Urine samples of prostate cancer and benign prostate hyperplasia patients	^{1}H NMR	Differential metabolite abundance analysis	Metabolites involved in pathways such as glycolysis and urea cycle are diminished in urine samples of cancer patients compared to benign hyperplasia patients, suggesting reduction waste for maximum anabolic utilization in prostate cancer
Li et al. 2020	Metabolomics	Plasma samples of Triple-negative breast cancer patients	LC-MS	Differential metabolite abundance analysis	Exploration of metabolic landscape of triple-negative breast cancer and development of a biomarker panel predictive of 5 year survival rates of patients
Mertins et al. 2016	Multi-omics	Breast cancer	WES, RNA-Seq, LC-MS/MS	Identification of SVs and CNVs, Proteogenomics analysis, PTM analysis, Clustering and network analysis	Providing insights into genomic alterations and the proteomics landscape and signaling networks of breast cancer. Identification of potential druggable targets
Vasaikar et al. 2019	Multi-omics	Colon cancer	WES, RNA-Seq, miRNA-Seq, LC-MS/MS, SRM	Identification of SVs and CNVs, Proteogenomics analysis, PTM analysis, Microsatellite instability prediction, Differential protein abundance analysis, Subtype prediction, Network analysis	Prioritization of somatic mutations for therapeutic purposes. Contribution to delineation of biology of colon cancer. Providing rationale for specific therapeutic interventions
Mun et al. 2019	Multi-omics	Tissue, blood samples, and cell lines of human gastric cancer	WES, RNA-Seq, LC-MS/MS	Microsatellite instability analysis, Germline and somatic variant identification, Proteogenomics analysis, Fusion gene identification, PTM analysis, Network analysis	Linking mutations and gene fusions to proteomics alterations. Candidate oncogene and tumor suppressor identification which can serve as prognostic biomarkers. Identification and characterization of diffuse gastric cancer subtypes
Campbell et al. 2020	Multi-omics	Pan-cancer	WGS, RNA-Seq	Germline and SV analysis, CNV analysis, Gene expression analysis	Providing critical insights to the mutational landscape of tumors
Ben-David et al. 2018	Multi-omics	cancer cell lines	WGS, Targeted exon sequencing, L1000 assay, Single-cell RNA-Seq	SV analysis, CNV analysis, Gene expression analysis	Confirmed rapid clonal evolution and increase in heterogeneity of cancer cell-lines with enormous effect on gene expression and treatment susceptibility
Davis et al. 2020	Multi-omics	PDX models of breast cancer	Single-cell RNA-Seq, LC-HRMS	Clustering and Differential expression/abundance analysis	Contribution to the delineation of the role of metabolism and particularly oxidative phosphorylation in tumor metastasis
Xu et al. 2020	Multi-omics	Lung adenocarcinoma	LC-MS/MS, WES, RNA-seq	CNV and SV analysis, Variant peptide analysis, Differential protein abundance analysis, Clustering analysis, PTM analysis, Fusion gene and alternative splicing analysis	Cancer subtype identification, Potential prognostic biomarker and therapeutic target discovery
Satpathy et al. 2020	Multi-omics	PDX models of breast cancer	WES, RNA-Seq, LC-MS/MS, PRM	SV and CNV analysis, Proteogenomics analysis, PTM analysis	Demonstration of feasibility of microscaled biopsies for proteogenomics analysis. Identification of potential treatment resistance mechanisms
Champion et al. 2018	Multi-omics	Pan-cancer	Microarray, RNA-Seq, Methylation assay	Network-based integrative analysis	Identification of pan-cancer driver genes involved in smoking-induced cancers and immune response
Sadeghi et al. 2020	Multi-omics	MCF7 breast cancer cell line	RNA-Seq, LC-MS/MS	Network-based integrative analysis	Identification of potential proteins involved in acid adaptation during epithelial-mesenchymal transition of tumors. Identification of potential prognostic biomarker
Taber et al. 2020	Multi-omics	Muscle-invasive bladder cancer	WES, RNA-Seq, Methylation arrays	SV and CNV analysis, Microsatellite instability analysis, Subtype analysis	Identification of combinative subtypes that can potential direct treatment decisions
Huang et al. 2021	Multi-omics	HPV-negative head and neck squamous cell carcinoma	WES, WGS, RNA-Seq, miRNA-Seq, methylation arrays, LC-MS/MS, Data-independent proteomics	Somatic and germline variant analysis, SV and CNV analysis, Gene fusion analysis, Differential protein abundance analysis, PTM analysis, proteogenomics analysis, Network and pathway analysis, Mutation prioritization, Neoantigen identification, Clustering and subtype analysis	Contribution to delineation of biology of head and neck squamous cell carcinoma. Identification of tumor subtypes for patient stratification and precision therapy. Identification of potential drug response biomarker.
Paull et al. 2021	Multi-omics	Pan-cancer	Mutation profiles and gene expression data from TCGA	Network-based integrative analysis	Identification of 24 master regulator modules of gene expression across cancer types. Identification of possible stratification strategy for precision medicine

WGS: Whole genome sequencing; WES: Whole exome sequencing; SV: Somatic variant; CNV: Copy number variation; PDX: Patient-derived xenograft; LC: Liquid chromatography; MS: Mass spectrometry; SRM: Selected reaction monitoring; PRM: Parallel reaction monitoring

References

Alexandrov, Ludmil B., Jaegil Kim, Nicholas J. Haradhvala, Mi Ni Huang, Alvin Wei Tian Ng, Yang Wu, Arnoud Boot, et al. 2020. "The Repertoire of Mutational Signatures in Human Cancer." *Nature* 578 (7793): 94–101. https://doi.org/10.1038/s41586-020-1943-3.

Alexandrov, Ludmil B., Serena Nik-Zainal, David C. Wedge, Samuel A.J.R. Aparicio, Sam Behjati, Andrew V. Biankin, Graham R. Bignell, et al. 2013. "Signatures of Mutational Processes in Human Cancer." *Nature* 500 (7463): 415–21. https://doi.org/10.1038/nature12477.

Bartoschek, Michael, Nikolay Oskolkov, Matteo Bocci, John Lövrot, Christer Larsson, Mikael Sommarin, Chris D. Madsen, et al. 2018. "Spatially and Functionally Distinct Subclasses of Breast Cancer-Associated Fibroblasts Revealed by Single Cell RNA Sequencing." *Nature Communications* 9 (1). https://doi.org/10.1038/s41467-018-07582-3.

Ben-David, Uri, Benjamin Siranosian, Gavin Ha, Helen Tang, Yaara Oren, Kunihiko Hinohara, Craig A. Strathdee, et al. 2018. "Genetic and Transcriptional Evolution Alters Cancer Cell Line Drug Response." *Nature* 560 (7718): 325–30. https://doi.org/10.1038/s41586-018-0409-3.

Bouchal, Pavel, Monika Dvořáková, Theodoros Roumeliotis, Zbyněk Bortlíček, Ivana Ihnatová, Iva Procházková, Jenny T.C. Ho, et al. 2015. "Combined Proteomics and Transcriptomics Identifies Carboxypeptidase B1 and Nuclear Factor KB (NF-KB) Associated Proteins as Putative Biomarkers of Metastasis in Low Grade Breast Cancer." *Molecular & Cellular Proteomics* 14 (7): 1814–30. https://doi.org/10.1074/mcp.M114.041335.

Bruzzone, Chiara, Ana Loizaga-Iriarte, Pilar Sánchez-Mosquera, Rubén Gil-Redondo, Ianire Astobiza, Tammo Diercks, Ana R. Cortazar, et al. 2020. "1 H NMR-Based Urine Metabolomics Reveals Signs of Enhanced Carbon and Nitrogen Recycling in Prostate Cancer." *Journal of Proteome Research* 19 (6): 2419–28. https://doi.org/10.1021/acs.jproteome.0c00091.

Campbell, Peter J., Gad Getz, Jan O. Korbel, Joshua M. Stuart, Jennifer L. Jennings, Lincoln D. Stein, Marc D. Perry, et al. 2020. "Pan-Cancer Analysis of Whole Genomes." *Nature*. https://doi.org/10.1038/s41586-020-1969-6.

Champion, Magali, Kevin Brennan, Tom Croonenborghs, Andrew J. Gentles, Nathalie Pochet, and Olivier Gevaert. 2018. "Module Analysis Captures Pancancer Genetically and Epigenetically Deregulated Cancer Driver Genes for Smoking and Antiviral Response." *EBioMedicine* 27: 156–66. https://doi.org/10.1016/j.ebiom.2017.11.028.

Davis, Ryan T., Kerrigan Blake, Dennis Ma, Mari B.Ishak Gabra, Grace A. Hernandez, Anh T. Phung, Ying Yang, et al. 2020. "Transcriptional Diversity and Bioenergetic Shift in Human Breast Cancer Metastasis Revealed by Single-Cell RNA Sequencing." *Nature Cell Biology* 22 (3): 310–20. https://doi.org/10.1038/s41556-020-0477-0.

Eswaran, Jeyanthy, Anelia Horvath, Sucheta Godbole, Sirigiri Divijendra Reddy, Prakriti Mudvari, Kazufumi Ohshiro, Dinesh Cyanam, et al. 2013. "RNA Sequencing of Cancer Reveals Novel Splicing Alterations." *Scientific Reports* 3. https://doi.org/10.1038/srep01689.

Guerreiro, Eduarda M., Reidun Øvstebø, Bernd Thiede, Daniela Elena Costea, Tine M. Søland, and Hilde Kanli Galtung. 2020. "Cancer Cell Line-Specific Protein Profiles in Extracellular Vesicles Identified by Proteomics." Edited by Arie Horowitz. *PLOS ONE* 15 (9): e0238591. https://doi.org/10.1371/journal.pone.0238591.

Huang, Chen, Lijun Chen, Sara R. Savage, Rodrigo Vargas Eguez, Yongchao Dou, Yize Li, Felipe da Veiga Leprevost, et al. 2021. "Proteogenomic Insights into the Biology and Treatment of HPV-Negative Head and Neck Squamous Cell Carcinoma." *Cancer Cell* 39 (3): 361-379.e16. https://doi.org/10.1016/j.ccell.2020.12.007.

Itamochi, Hiroaki, Tetsuro Oishi, Nao Oumi, Satoshi Takeuchi, Kosuke Yoshihara, Mikio Mikami, Nobuo Yaegashi, et al. 2017. "Whole-Genome Sequencing Revealed Novel Prognostic Biomarkers and Promising Targets for Therapy of Ovarian Clear Cell Carcinoma." *British Journal of Cancer* 117 (5): 717–24. https://doi.org/10.1038/bjc.2017.228.

Li, Lixian, Xiaodong Zheng, Qi Zhou, Nathaniel Villanueva, Weiqi Nian, Xingming Liu, and Tao Huan. 2020. "Metabolomics-Based Discovery of Molecular Signatures for Triple Negative Breast Cancer in Asian Female Population." *Scientific Reports* 10 (1): 370. https://doi.org/10.1038/s41598-019-57068-5.

Luo, Xialin, Jingjing Liu, Huaizhi Wang, and Haitao Lu. 2020. "Metabolomics Identified New Biomarkers for the Precise Diagnosis of Pancreatic Cancer and Associated Tissue Metastasis." *Pharmacological Research* 156 (June): 104805. https://doi.org/10.1016/j.phrs.2020.104805.

Mertins, Philipp, D. R. Mani, Kelly V. Ruggles, Michael A. Gillette, Karl R. Clauser, Pei Wang, Xianlong Wang, et al. 2016. "Proteogenomics Connects Somatic Mutations to Signalling in Breast Cancer." *Nature* 534 (7605): 55–62. https://doi.org/10.1038/nature18003.

Mun, Dong-Gi, Jinhyuk Bhin, Sangok Kim, Hyunwoo Kim, Jae Hun Jung, Yeonjoo Jung, Ye Eun Jang, et al. 2019. "Proteogenomic Characterization of Human Early-Onset Gastric Cancer." *Cancer Cell* 35 (1): 111-124.e10. https://doi.org/10.1016/j.ccell.2018.12.003.

Panou, Vasiliki, Meghana Gadiraju, Arthur Wolin, Caroline M. Weipert, Emily Skarda, Aliya N. Husain, Jyoti D. Patel, et al. 2018. "Frequency of Germline Mutations in Cancer Susceptibility Genes in Malignant Mesothelioma." *Journal of Clinical Oncology* 36 (28): 2863–71. https://doi.org/10.1200/JCO.2018.78.5204.

Patel, Anoop P., Itay Tirosh, John J. Trombetta, Alex K. Shalek, Shawn M. Gillespie, Hiroaki Wakimoto, Daniel P. Cahill, et al. 2014. "Single-Cell RNA-Seq Highlights Intratumoral Heterogeneity in Primary Glioblastoma." *Science*. https://doi.org/10.1126/science.1254257.

Paull, Evan O., Alvaro Aytes, Sunny J. Jones, Prem S. Subramaniam, Federico M. Giorgi, Eugene F. Douglass, Somnath Tagore, et al. 2021. "A Modular Master Regulator Landscape Controls Cancer Transcriptional Identity." *Cell* 184 (2): 334-351.e20. https://doi.org/10.1016/j.cell.2020.11.045.

Procházková, Iva, Juraj Lenčo, and Pavel Bouchal. 2017. "Targeted Proteomics Driven Verification of Biomarker Candidates Associated with Breast Cancer Aggressiveness." In , 177–84. https://doi.org/10.1007/7651_2017_111.

Sadeghi, Mehdi, Bryce Ordway, Ilyia Rafiei, Punit Borad, Bin Fang, John L. Koomen, Chaomei Zhang, Sean Yoder, Joseph Johnson, and Mehdi Damaghi. 2020. "Integrative Analysis of Breast Cancer Cells Reveals an Epithelial-Mesenchymal Transition Role in Adaptation to Acidic Microenvironment." *Frontiers in Oncology* 10 (March): 1–14. https://doi.org/10.3389/fonc.2020.00304.

Satpathy, Shankha, Eric J. Jaehnig, Karsten Krug, Beom Jun Kim, Alexander B. Saltzman, Doug W. Chan, Kimberly R. Holloway, et al. 2020. "Microscaled Proteogenomic Methods for Precision Oncology." *Nature Communications* 11 (1). https://doi.org/10.1038/s41467-020-14381-2.

Swiatly, Agata, Agnieszka Horala, Jan Matysiak, Joanna Hajduk, Ewa Nowak-Markwitz, and Zenon Kokot. 2018. "Understanding Ovarian Cancer: ITRAQ-Based Proteomics for Biomarker Discovery." *International Journal of Molecular Sciences* 19 (8): 2240. https://doi.org/10.3390/ijms19082240.

Taber, Ann, Emil Christensen, Philippe Lamy, Iver Nordentoft, Frederik Prip, Sia Viborg Lindskrog, Karin Birkenkamp-Demtröder, et al. 2020. "Molecular Correlates of Cisplatin-Based Chemotherapy Response in Muscle Invasive Bladder Cancer by Integrated Multi-Omics Analysis." *Nature Communications* 11 (1): 4858. https://doi.org/10.1038/s41467-020-18640-0.

Vasaikar, Suhas, Chen Huang, Xiaojing Wang, Vladislav A. Petyuk, Sara R. Savage, Bo Wen, Yongchao Dou, et al. 2019. "Proteogenomic Analysis of Human Colon Cancer Reveals New Therapeutic Opportunities." *Cell* 177 (4): 1035-1049.e19. https://doi.org/10.1016/j.cell.2019.03.030.

Xu, Jun-Yu, Chunchao Zhang, Xiang Wang, Linhui Zhai, Yiming Ma, Yousheng Mao, Kun Qian, et al. 2020. "Integrative Proteomic Characterization of Human Lung Adenocarcinoma." *Cell* 182 (1): 245-261.e17. https://doi.org/10.1016/j.cell.2020.05.043.

Yamada, Atsushi, Pingjian Yu, Wei Lin, Yoshinaga Okugawa, C. Richard Boland, and Ajay Goel. 2018. "A RNA-Sequencing Approach for the Identification

of Novel Long Non-Coding RNA Biomarkers in Colorectal Cancer." *Scientific Reports* 8 (1): 2–11. https://doi.org/10.1038/s41598-017-18407-6.

Zhou, Bin, Zhe Zhou, Yuling Chen, Haiteng Deng, Yunlong Cai, Xiaolong Rao, Yuxin Yin, and Long Rong. 2020. "Plasma Proteomics-Based Identification of Novel Biomarkers in Early Gastric Cancer." *Clinical Biochemistry* 76 (February): 5–10. https://doi.org/10.1016/j.clinbiochem.2019.11.001.

Zhu, Mingzhe, Yanqi Dang, Zhenhua Yang, Yang Liu, Li Zhang, Yangxian Xu, Wenjun Zhou, and Guang Ji. 2020. "Comprehensive RNA Sequencing in Adenoma-Cancer Transition Identified Predictive Biomarkers and Therapeutic Targets of Human CRC." *Molecular Therapy - Nucleic Acids* 20 (June): 25–33. https://doi.org/10.1016/j.omtn.2020.01.031.
