# Supplementary material for: Prospects and challenges of cancer systems medicine: from genes to disease networks
Source: Brief Bioinform. 2021 Sep 1;23(1):bbab343. doi: 10.1093/bib/bbab343 (PMC8769701; doi:10.1093/bib/bbab343)
Supplement: Supplementary_Table_S2_bbab343 [file supplementary_table_s2_bbab343.pdf]

Table S2 A list of some of the prominent tools utilized in integration of multi-modal data and their comparative performance

Tool	Implementation	Description	Comparative performance	Reference
CC[ϕ]	R	clustering of samples through assessment of pairwise similarity of samples via repeated runs of subsampling and clustering	ϕ: One of the three worst performing algorithms in recovering the true number of clusters. One of the five best performing algorithms in clustering.	Wilkerson and Hayes 2010
CIMLR[ɫ ϕ]	R/MATLAB	Cancer subtyping through multi-omics data integration via multi-kernel learning	ɫ: Moderate performance in clustering of pan-cancer types<br>ϕ: One of the three worst performing algorithms in recovering the true number of clusters. Good performance in clustering.	Ramazzotti et al. 2018
iCluster[¥ ɫ]	R	Dimensionality reduction of multi-omics data and sample clustering through Gaussian latent variable modeling of intra- and inter-omics relationships	¥: Performed second to intNMF in identifying simulated multi-omics sample clusters. Performed worst in clustering of single-cell samples<br>ɫ: Second best performing tool (after SNF) in clustering pan-cancer types	Shen, Olshen, and Ladanyi 2009
iClusterPlus[ϕ]	R	An enhancement of iCluster that is able to integrate binary, continuous, and categorical data	ϕ: One of the two slowest methods. One of the five best performing tools in recovering the true number of clusters. Good performance in clustering.	Mo et al. 2013
intNMF[¥ ϕ]	R	Dimensionality reduction of multi-omics and sample clustering based on non-negative matrix factorization	¥: Performed flawless in identifying simulated multi-omics sample clusters. Best performing tool for clustering purposes. Not recommended for other tasks such as association with cellular pathways and biomarker discovery.<br>ϕ: One of the two slowest methods. Good performance in recovering the true number of clusters. Good performance in clustering	Chalise and Fridley 2017
JIVE[¥ †]	R	Dimensionality reduction of multi-omics data through an extension of principal component analysis. Can handle missing samples	¥: One of the four best performing tools in survival analysis and clinical annotation. Performed poor in single-cell clustering<br>†: Overall poor performance. Least resistant algorithm to noise and complexity. Only suitable for extracting shared signals. Feature selection did not improve the performance of this method	Lock et al. 2013
LRACluster[ɫ ϕ]	R	Dimensionality reduction and clustering of multi-omics data through probabilistic modelling	ɫ: Worst performance in clustering of pan-cancer types<br>ϕ: One of the five best performing tools in recovering the true number of clusters. One of the five best performing algorithms in clustering.	Wu et al. 2015
MCCA[†]	R	An extension to canonical correlation analysis for multiple data types	†: Moderate performance with increase in complexity. Only suitable for extracting complementary signals. Feature selection improved performance of this algorithm the most among the evaluated methods	Witten and Tibshirani 2009
MCIA[¥ ϕ †]	R	Dimensionality reduction of multi-omics data through isolated factorization of each omics and subsequent maximization of co-inertia	¥: Consistently-well performing across all benchmarks. Recommended as the overall best performing method in this study<br>ϕ: One of the three worst performing algorithms in recovering the true number of clusters. Poor performance in clustering<br>†: Moderate classification performance with increase in complexity. Suitable for extracting both shared and complementary signals	Meng et al. 2014
MFA[†]	R	Dimensionality reduction of multi-omics data through a modification (utilization of co-inertia) of generalized canonical correlation	†: Second to SNF in clustering performance and resistance to noise as complexity increased. Suitable for extracting both shared and complementary signals	de Tayrac et al. 2009
mixKernel[ϕ]	R	A kernel-based framework where multiple kernels from different multi-omics data are integrated to form a meta-kernel which can subsequently be interpreted through various approaches such as PCA-based methods	ϕ: Good performance in recovering the true number of clusters. Good performance in clustering.	Mariette and Villa-Vialaneix 2018
moCluster[ϕ]	R	Dimensionality reduction and clustering of multi-omics data through a multiblock multivariate analysis	ϕ: One of the five best performing tools in recovering the true number of clusters. One of the five best performing algorithms in clustering.	Meng et al. 2016
MOFA[¥ ϕ]	R	Dimensionality reduction through Bayesian factor analysis. Can handle missing samples	¥: One of the four best performing tools in survival analysis and clinical annotation. Moderate performance in clustering of single-cell samples. The only tool that was capable of identifying slightly nonlinear signals in this study<br>ϕ: Faced difficulties in providing results in the framework of this study and thus was not considered in the evaluations of the original comparative study	Argelaguet et al. 2018
MSFA[¥]	R	Dimensionality reduction of multi-omics data through Bayesian factor analysis	¥: One of the two best performing tools for clustering of single-cell samples	De Vito et al. 2019
PINS[ɫ]	R	Multi-omics data integration by pair-wise connectivity matrix analysis. Requires both original omics data and clustering results	ɫ: Poor performance in clustering of pan-cancer types	T. Nguyen et al. 2017
PINSPlus[ϕ]	R	An optimization of PINS algorithms	ϕ: One of the five best performing tools in recovering the true number of clusters. One of the five best performing algorithms in clustering.	H. Nguyen et al. 2019
RGCCA[¥ ϕ]	R	An extension to canonical correlation analysis for dimensionality reduction of multi-omics data through isolated factorization of each omics and subsequent maximization of correlation	¥: One of the four best performing tools in survival analysis and clinical annotation. Moderate performance in clustering of single-cell samples<br>ϕ: Poor performance in recovering the true number of clusters. Poor performance in clustering	M. Tenenhaus, Tenenhaus, and Groenen 2017
SGCCA[ϕ]	R	An extension to RGCCA solving the issue of selection of variables from each block	ϕ: One of the five best performing tools in recovering the true number of clusters. Good performance in clustering	A. Tenenhaus et al. 2014
SNF[ɫ ϕ †]	R/MATLAB	Network-based Integration of multi-omics data through fusion of multiple sample-based omics-specific network into a single similarity network	ɫ: Performed best in clustering pan-cancer types<br>ϕ: Good performance in recovering the true number of clusters. The fastest algorithm. One of the five best performing algorithms in clustering and the overall best method for classification of individuals.<br>†: The overall best performing algorithm for classification in this study. The most resistant algorithm to noise and increase in complexity	Wang et al. 2014
Scikit-fusion[¥]	Python	Data fusion through matrix tri-factorization. Can incorporate additional information in the factorization process. Capable of handling missing samples	¥: Performed poor in identifying simulated multi-omics sample clusters. Moderate performance in clustering of single-cell samples. It should be noted that the ability of Scikit-fusion for incorporation of additional information such as various annotations was not exploited in this study	Zitnik and Zupan 2015
tICA[¥]	R	Extension of independent component analysis for integration of multi-omics data	¥: One of the two best performing tools for clustering of single-cell samples. Performed efficiently in biological annotation. Performed poor in identifying simulated multi-omics sample clusters	Teschendorff et al. 2018

Superscript symbols indicate inclusion of the tool in the respective study; ¥: (Cantini et al. 2021); ɫ: (Wei et al. 2021); ϕ: (Pierre-Jean et al. 2020); †: (Tini et al. 2019)

References

Argelaguet, Ricard, Britta Velten, Damien Arnol, Sascha Dietrich, Thorsten Zenz, John C Marioni, Florian Buettner, Wolfgang Huber, and Oliver Stegle. 2018. "Multi- Omics Factor Analysis—a Framework for Unsupervised Integration of Multi-omics Data Sets." *Molecular Systems Biology* 14 (6): 1–13. https://doi.org/10.15252/msb.20178124.

Cantini, Laura, Pooya Zakeri, Celine Hernandez, Aurelien Naldi, Denis Thieffry, Elisabeth Remy, and Anaïs Baudot. 2021. "Benchmarking Joint Multi-Omics Dimensionality Reduction Approaches for the Study of Cancer." *Nature Communications* 12 (1): 124. https://doi.org/10.1038/s41467-020-20430-7.

Chalise, Prabhakar, and Brooke L. Fridley. 2017. "Integrative Clustering of Multi-Level 'omic Data Based on Non-Negative Matrix Factorization Algorithm." Edited by Shyamal D Peddada. *PLOS ONE* 12 (5): e0176278. https://doi.org/10.1371/journal.pone.0176278.

Lock, Eric F., Katherine A. Hoadley, J. S. Marron, and Andrew B. Nobel. 2013. "Joint and Individual Variation Explained (JIVE) for Integrated Analysis of Multiple Data Types." *The Annals of Applied Statistics* 7 (1). https://doi.org/10.1214/12-AOAS597.

Mariette, Jérôme, and Nathalie Villa-Vialaneix. 2018. "Unsupervised Multiple Kernel Learning for Heterogeneous Data Integration." Edited by Jonathan Wren. *Bioinformatics* 34 (6): 1009–15. https://doi.org/10.1093/bioinformatics/btx682.

Meng, Chen, Dominic Helm, Martin Frejno, and Bernhard Kuster. 2016. "MoCluster: Identifying Joint Patterns Across Multiple Omics Data Sets." *Journal of Proteome Research* 15 (3): 755–65. https://doi.org/10.1021/acs.jproteome.5b00824.

Meng, Chen, Bernhard Kuster, Aedín C Culhane, and Amin Gholami. 2014. "A Multivariate Approach to the Integration of Multi-Omics Datasets." *BMC Bioinformatics* 15 (1): 162. https://doi.org/10.1186/1471-2105-15-162.

Mo, Qianxing, Sijian Wang, Venkatraman E. Seshan, Adam B. Olshen, Nikolaus Schultz, Chris Sander, R. Scott Powers, Marc Ladanyi, and Ronglai Shen. 2013. "Pattern Discovery and Cancer Gene Identification in Integrated Cancer Genomic Data." *Proceedings of the National Academy of Sciences* 110 (11): 4245–50. https://doi.org/10.1073/pnas.1208949110.

Nguyen, Hung, Sangam Shrestha, Sorin Draghici, and Tin Nguyen. 2019. "PINSPlus: A Tool for Tumor Subtype Discovery in Integrated Genomic Data." Edited by Bonnie Berger. *Bioinformatics* 35 (16): 2843–46. https://doi.org/10.1093/bioinformatics/bty1049.

Nguyen, Tin, Rebecca Tagett, Diana Diaz, and Sorin Draghici. 2017. "A Novel Approach for Data Integration and Disease Subtyping." *Genome Research* 27 (12): 2025–39. https://doi.org/10.1101/gr.215129.116.

Pierre-Jean, Morgane, Jean-François Deleuze, Edith Le Floch, and Florence Mauger. 2020. "Clustering and Variable Selection Evaluation of 13 Unsupervised Methods for Multi-Omics Data Integration." *Briefings in Bioinformatics* 21 (6): 2011–30. https://doi.org/10.1093/bib/bbz138.

Ramazzotti, Daniele, Avantika Lal, Bo Wang, Serafim Batzoglou, and Arend Sidow. 2018. "Multi-Omic Tumor Data Reveal Diversity of Molecular Mechanisms That Correlate with Survival." *Nature Communications* 9 (1): 4453. https://doi.org/10.1038/s41467-018-06921-8.

Shen, Ronglai, Adam B. Olshen, and Marc Ladanyi. 2009. "Integrative Clustering of Multiple Genomic Data Types Using a Joint Latent Variable Model with Application to Breast and Lung Cancer Subtype Analysis." *Bioinformatics* 25 (22): 2906–12. https://doi.org/10.1093/bioinformatics/btp543.

Tayrac, Marie de, Sebastien Le, Marc Aubry, Jean Mosser, and Francois Husson. 2009. "Simultaneous Analysis of Distinct Omics Data Sets with Integration of Biological Knowledge: Multiple Factor Analysis Approach." *BMC Genomics* 10 (1): 32. https://doi.org/10.1186/1471-2164-10-32.

Tenenhaus, A., C. Philippe, V. Guillemot, K.-A. Le Cao, J. Grill, and V. Frouin. 2014. "Variable Selection for Generalized Canonical Correlation Analysis." *Biostatistics* 15 (3): 569–83. https://doi.org/10.1093/biostatistics/kxu001.

Tenenhaus, Michel, Arthur Tenenhaus, and Patrick J. F. Groenen. 2017. "Regularized Generalized Canonical Correlation Analysis: A Framework for Sequential Multiblock Component Methods." *Psychometrika* 82 (3): 737–77. https://doi.org/10.1007/s11336-017-9573-x.

Teschendorff, Andrew E., Han Jing, Dirk S. Paul, Joni Virta, and Klaus Nordhausen. 2018. "Tensorial Blind Source Separation for Improved Analysis of Multi-Omic Data." *Genome Biology* 19 (1): 76. https://doi.org/10.1186/s13059-018-1455-8.

Tini, Giulia, Luca Marchetti, Corrado Priami, and Marie-Pier Scott-Boyer. 2019. "Multi-Omics Integration—a Comparison of Unsupervised Clustering Methodologies." *Briefings in Bioinformatics* 20 (4): 1269–79. https://doi.org/10.1093/bib/bbx167.

Vito, Roberta De, Ruggero Bellio, Lorenzo Trippa, and Giovanni Parmigiani. 2019. "Multi-study Factor Analysis." *Biometrics* 75 (1): 337–46. https://doi.org/10.1111/biom.12974.

Wang, Bo, Aziz M Mezlini, Feyyaz Demir, Marc Fiume, Zhuowen Tu, Michael Brudno, Benjamin Haibe-Kains, and Anna Goldenberg. 2014. "Similarity Network Fusion for Aggregating Data Types on a Genomic Scale." *Nature Methods* 11 (3): 333–37. https://doi.org/10.1038/nmeth.2810.

Wei, Zhuohui, Yue Zhang, Wanlin Weng, Jiazhou Chen, and Hongmin Cai. 2021. "Survey and Comparative Assessments of Computational Multi-Omics Integrative Methods with Multiple Regulatory Networks Identifying Distinct Tumor Compositions across Pan-Cancer Data Sets." *Briefings in Bioinformatics* 22 (3). https://doi.org/10.1093/bib/bbaa102.

Wilkerson, Matthew D., and D. Neil Hayes. 2010. "ConsensusClusterPlus: A Class Discovery Tool with Confidence Assessments and Item Tracking." *Bioinformatics* 26 (12): 1572–73. https://doi.org/10.1093/bioinformatics/btq170.

Witten, Daniela M, and Robert J. Tibshirani. 2009. "Extensions of Sparse Canonical Correlation Analysis with Applications to Genomic Data." *Statistical Applications in Genetics and Molecular Biology* 8 (1): 1–27. https://doi.org/10.2202/1544-6115.1470.

Wu, Dingming, Dongfang Wang, Michael Q. Zhang, and Jin Gu. 2015. "Fast Dimension Reduction and Integrative Clustering of Multi-Omics Data Using Low-Rank Approximation: Application to Cancer Molecular Classification." *BMC Genomics* 16 (1): 1022. https://doi.org/10.1186/s12864-015-2223-8.

Zitnik, Marinka, and Blaz Zupan. 2015. "Data Fusion by Matrix Factorization." *IEEE Transactions on Pattern Analysis and Machine Intelligence* 37 (1): 41–53. https://doi.org/10.1109/TPAMI.2014.2343973.
