# Supplementary material for: Prospects and challenges of cancer systems medicine: from genes to disease networks
Source: Brief Bioinform. 2021 Sep 1;23(1):bbab343. doi: 10.1093/bib/bbab343 (PMC8769701; doi:10.1093/bib/bbab343)
Supplement: Supplementary_Table_S3_bbab343 [file supplementary_table_s3_bbab343.pdf]

Table S3 A list of some of the prominent methods utilized for the enrichment analysis and their comparative performance

Method	Description	Comparative performance	Reference
**CAMERA[ϕ]**	A competitive gene set testing method for enrichment analysis that does not incorporate the interconnectedness information but adjusts the parametric or rank-based test based on the estimated variance inflation associated with inter-gene correlation	ϕ: Well-performing for the analysis of genomics data. Poor performance for the analysis of metabolomics data	Wu and Smyth 2012
**CePaGSA†[ǂ]**	FCS implementation of CePa method	†: Reported 112 out of 150 pathways as false-positives and 0 out of 150 pathways as false-negatives under the null hypothesis. Had the lowest p-value for true pathways. Was not considered in AUC assessment.<br>ǂ: Demonstrated the best sensitivity and by far the worst specificity and accuracy for the analysis of overlapping pathways. When analyzing non-overlapping pathways, it demonstrated moderate sensitivity, and was among the tools with best specificity and accuracy (topology-aware tools). Similar to the results in †, this tool by far exhibited the lowest p-value for the target genes which is probably due to its low specificity. Demonstrated poor performance in ranking the target pathways.	Gu et al. 2012
**CePaORA†[ǂ ϕ ¥]**	CePa is an enrichment method that incorporates topology information through a user-specified centrality parameter. CePaORA is the ORA implementation of this method	†: Reported 16 out of 150 pathways as false-positives and 75 out of 150 pathways as false-negatives under the null hypothesis. Third lowest p-value for true pathways. Was not considered in AUC assessment.<br>ǂ: Demonstrated good sensitivity, moderate specificity and accuracy for the analysis of overlapping pathways. When analyzing non-overlapping pathways, it demonstrated moderate sensitivity, and was among the tools with best specificity and accuracy (topology-aware tools). Demonstrated poor performance in ranking the target pathways.<br>ϕ: Poor performance for the analysis of genomics data similar to all other ORA-based tested tools. Poor performance in the analysis of metabolomics data<br>¥: Along with SPIA, demonstrated the best balance between consistency and the number of identified significant pathway when analyzing cancer datasets. However, the consistency was only achieved when the data was scaled	Gu et al. 2012
**DAVID[¥]**	A modification of Fisher's exact test through jackknifing to favor significance of categories with a higher number of differential molecules supporting it and restrict the effect of possible false-positive significant molecules	¥: Was unable to provide consistent results	Jiao et al. 2012
**DEGraph[ϕ]**	A topology-aware enrichment method based on direct assessment of differential expression of gene networks consistent with the pathway structure through multivariate two-sample tests of means	ϕ: Outperformed all the other tested algorithms for both genomics and metabolomics analyses and was concluded to be the most overall robust algorithm in this study	Jacob, Neuvial, and Dudoit 2012
**Fisher's exact test†[ǂ]**	A non-parametric test that determines whether association of two lists occurred randomly	†: Reported highest number of false-positives (137 out of 150 pathways) and 0 false-negatives under the null condition. Demonstrated the second worst AUC among the evaluated methods<br>ǂ: Demonstrated poor sensitivity, moderate specificity and accuracy for the analysis of overlapping pathways. When analyzing non-overlapping pathways, demonstrated the worst sensitivity, good specificity and accuracy (the best among the methods that were not topology-aware). Demonstrated poor performance in ranking the target pathways. Calculated the highest p-value for the target pathways.	Fisher 1992
**GOstats†**	Identifies the enriched list of molecules through classical or conditional hypergeometric test	†: Reported 54 out of 150 pathways as false-positives and 33 out of 150 pathways as false-negatives under the null hypothesis.	Falcon and Gentleman 2007
**GSA†**	An extension to GSEA that utilizes "maxmean" for enrichment score calculation	†: Reported 78 out of 150 pathways as false-positives and 28 out of 150 pathways as false-negatives under the null hypothesis.	Efron and Tibshirani 2007
**GSEA†[¥]**	An FCS method that determines if distribution of molecules in a list that are present in a reference list is non-random (i.e. if there is a high density of molecules present in the reference list either in top or bottom of an input sorted list)	†: Reported no false-positives or false-negatives under the null. Has high specificity but suffers from low sensitivity. Had the highest AUC among the tested methods that were not topology-aware<br>¥: Was unable to provide consistent results	Subramanian et al. 2005
**Kolmogorov-Smirnov test†[ǂ]**	A non-parametric test that determines the distance between the empirical distributions of molecules in a given list that are either present or absent in a reference list	†: Reported 64 out of 150 pathways as false-positives and 2 out of 150 pathways as false-negatives under the null hypothesis. The worst performing method in ranking true pathways.<br>ǂ: Demonstrated good sensitivity, moderate specificity and accuracy for analysis of overlapping pathways. When analyzing non-overlapping pathways, demonstrated the best sensitivity and by far the worst specificity and accuracy.	Massey 1951
**NetGSA[ϕ ¥]**	A complex mathematical model for topology-aware enrichment analysis that can utilize a probabilistic graphical model that will complete the network information (for incomplete networks) under constraints of the available topology data	ϕ: Was concluded to be well-performing for the analysis of metabolomics data (The other tested methods were CAMERA, CePaORA, Pathnet, and the best performing DEGraph). Exhibited inflated false-positives<br>¥: Was unable to provide consistent results	Ma, Shojaie, and Michailidis 2016
**PADOG†**	An FCS enrichment method that attempts to reduce insignificant signals through down-weighting of ubiquitous molecules	†: Reported 71 out of 150 pathways as false-positives and 59 out of 150 pathways as false-negatives under the null hypothesis. Exhibited the best performance in ranking true pathways. Second lowest p-value for true pathways.	Tarca et al. 2012
**PathNet†[ǂ ϕ]**	A topology-aware enrichment method implementing a "guilt-by-association" strategy	†: Reported 0 out of 150 pathways as false-positives and the highest number of false-negatives (129 out of 150 pathways) under the null hypothesis. Had the highest p-value for true pathways. Was not considered in AUC assessment.<br>ǂ: Demonstrated poor sensitivity, and moderated specificity and accuracy for the analysis of the overlapping pathways. When analyzing non-overlapping pathways, it demonstrated good sensitivity (the best among the topology-aware tools) and was among the tools with best specificity and accuracy (topology-aware tools). Calculated the second lowest p-value for the target pathways and demonstrated the best performance in ranking the target pathways<br>ϕ: Second best performing tool for the analysis of genomics data. Poor performance in the analysis of metabolomics data	Dutta, Wallqvist, and Reifman 2012
**PRS[ϕ]**	A topology-aware algorithm for enrichment analysis that uses a pathway scoring system that incorporates gene expression data in the form of fold-change and the topology data in the form of node weights representing differential nodes downstream of the starting node	ϕ: Poor performance for the analysis of genomics data similar to all other ORA-based tested tools	Ibrahim et al. 2012
**Pathifier[¥]**	An enrichment method that scores deregulation of each given pathway for every perturbed sample. For each pathway, an n dimensional space (n being the number of genes in the given pathway) based on the expression levels of all the genes in that pathway is created, with each sample occupying one point in this space. Next, a nonlinear principal curve derived from variation of all the samples in the dimensional space is calculated. Finally, the deregulation score is the distance between the projection of each perturbed sample on this curve and the centroid of projection of normal samples	¥: The results were consistent but were unreliable since all pathways for all datasets (except for one pathway for a single dataset) was identified as significant. The number of false-positives in artificial datasets were also high	Drier, Sheffer, and Domany 2013
**ROntoTools†[ϕ]**	An implementation of impact analysis	†: Reported 68 out of 150 pathways as false-positives and 8 out of 150 pathways as false-negatives under the null hypothesis. Exhibited the highest AUC among the evaluated methods (note that the only other topology-aware for this assessment was SPIA and other topology-aware methods evaluated in this study were not compatible with this particular assessment)<br>ϕ: When used in the FCS mode, it was well-performing for the analysis of genomics data. The ORA mode exhibited poor performance similar to all other ORA-based tested tools	Voichita, Ansari, and Draghici 2021
**SPIA†[ǂ ϕ ¥]**	A topology-aware impact analysis-based enrichment method that combines the over-representation of molecules in a reference list with the pathway perturbation information based on expression changes	†: Reported 36 out of 150 pathways as false-positives and 55 out of 150 pathways as false-negatives under the null hypothesis.<br>ǂ: Demonstrated poor sensitivity, the second best specificity and accuracy for the analysis of overlapping pathways. When analyzing non-overlapping pathways. When analyzing non-overlapping pathways, it demonstrated moderate sensitivity, and was among the tools with best specificity and accuracy (topology-aware tools). Demonstrated poor performance in ranking the target pathways. Calculated the second highest p-value for the target pathways.<br>ϕ: Poor performance for the analysis of genomics data similar to all other ORA-based tested tools<br>¥: Along with CePaORA, demonstrated the best balance between consistency and the number of identified significant pathways when analyzing cancer datasets. Overall best performance in this study	Tarca et al. 2009
**topologyGSA[ϕ]**	A topology-aware method for enrichment analysis that derives a directed acyclic graph from the user-defined pathway and converts it to a moral graph by removing its directionalities and adding edges between parent nodes of each node. Subsequently it tests differential expression via multiple analysis of variance if the variances are equal or through Behrens-Fisher problem if they are not	ϕ: Well-performing for the analysis of genomics data	Massa, Chiogna, and Romualdi 2010
**WebGestalt†**	Identifies the enriched list of molecules through hypergeometric and Fisher's exact test	†: Reported 120 out of 150 pathways as false-positives and 8 out of 150 pathways as false-negatives under the null hypothesis. Exhibited the worst AUC among the evaluated methods	Wang et al. 2013
**Wilcoxon rank sum test†[ǂ]**	A non-parametric test that determines whether the difference between a parameter (e.g. p-value) calculated for two lists is statistically significant	†: Reported 114 out of 150 pathways as false-positives and 0 out of 150 pathways as false-negatives under the null hypothesis. Second worst performing method in ranking true pathways.<br>ǂ: Demonstrated good sensitivity, best specificity, and the best accuracy in analyzing the overlapping pathways. When analyzing non-overlapping pathways, it demonstrated second best sensitivity and second worst specificity and accuracy. Second best performance in ranking the target pathways.	Wilcoxon 1992

Superscripts indicate inclusion of the method in the respective study;
†: (Nguyen et al. 2019), it is notable that overall, the topology-aware tools outperformed other tools in every aspect of this study;
ǂ: (Bayerlová et al. 2015), Note that while this study has shown consistent superior accuracy of topology-aware tools for the analysis of non-overlapping pathways, it could not identify any topology-aware tool as superior to the tools that are not topology-aware in the more realistic scenarios (overlapping pathways);
ϕ: (Ma, Shojaie, and Michailidis 2019);
¥: (Jaakkola and Elo 2016), the reported results are based on the analysis of cancer and artificial datasets since when analyzing non-cancer (diabetes) datasets, all the methods exhibited overall poor performance

References

Bayerlová, Michaela, Klaus Jung, Frank Kramer, Florian Klemm, Annalen Bleckmann, and Tim Beißbarth. 2015. "Comparative Study on Gene Set and Pathway Topology-Based Enrichment Methods." *BMC Bioinformatics* 16 (1): 334. https://doi.org/10.1186/s12859-015-0751-5.

Drier, Y., M. Sheffer, and E. Domany. 2013. "Pathway-Based Personalized Analysis of Cancer." *Proceedings of the National Academy of Sciences* 110 (16): 6388–93. https://doi.org/10.1073/pnas.1219651110.

Dutta, Bhaskar, Anders Wallqvist, and Jaques Reifman. 2012. "PathNet: A Tool for Pathway Analysis Using Topological Information." *Source Code for Biology and Medicine* 7 (1): 10. https://doi.org/10.1186/1751-0473-7-10.

Efron, Bradley, and Robert Tibshirani. 2007. "On Testing the Significance of Sets of Genes." *The Annals of Applied Statistics* 1 (1). https://doi.org/10.1214/07-AOAS101.

Falcon, S., and R. Gentleman. 2007. "Using GOstats to Test Gene Lists for GO Term Association." *Bioinformatics* 23 (2): 257–58. https://doi.org/10.1093/bioinformatics/btl567.

Fisher, R. A. 1992. "Statistical Methods for Research Workers." In , 66–70. https://doi.org/10.1007/978-1-4612-4380-9_6.

Gu, Zuguang, Jialin Liu, Kunming Cao, Junfeng Zhang, and Jin Wang. 2012. "Centrality-Based Pathway Enrichment: A Systematic Approach for Finding Significant Pathways Dominated by Key Genes." *BMC Systems Biology* 6 (1): 56. https://doi.org/10.1186/1752-0509-6-56.

Ibrahim, Maysson Al-Haj, Sabah Jassim, Michael Anthony Cawthorne, and Kenneth Langlands. 2012. "A Topology-Based Score for Pathway Enrichment." *Journal of Computational Biology* 19 (5): 563–73. https://doi.org/10.1089/cmb.2011.0182.

Jaakkola, Maria K., and Laura L. Elo. 2016. "Empirical Comparison of Structure-Based Pathway Methods." *Briefings in Bioinformatics* 17 (2): 336–45. https://doi.org/10.1093/bib/bbv049.

Jacob, Laurent, Pierre Neuvial, and Sandrine Dudoit. 2012. "More Power via Graph-Structured Tests for Differential Expression of Gene Networks." *The Annals of Applied Statistics* 6 (2). https://doi.org/10.1214/11-AOAS528.

Jiao, X., B. T. Sherman, D. W. Huang, R. Stephens, M. W. Baseler, H. C. Lane, and R. A. Lempicki. 2012. "DAVID-WS: A Stateful Web Service to Facilitate Gene/Protein List Analysis." *Bioinformatics* 28 (13): 1805–6. https://doi.org/10.1093/bioinformatics/bts251.

Ma, Jing, Ali Shojaie, and George Michailidis. 2016. "Network-Based Pathway Enrichment Analysis with Incomplete Network Information." *Bioinformatics* 32 (20): 3165–74. https://doi.org/10.1093/bioinformatics/btw410.

———. 2019. "A Comparative Study of Topology-Based Pathway Enrichment Analysis Methods." *BMC Bioinformatics* 20 (1): 1–14. https://doi.org/10.1186/s12859-019-3146-1.

Massa, Maria Sofia, Monica Chiogna, and Chiara Romualdi. 2010. "Gene Set Analysis Exploiting the Topology of a Pathway." *BMC Systems Biology* 4 (1): 121. https://doi.org/10.1186/1752-0509-4-121.

Massey, Frank J. 1951. "The Kolmogorov-Smirnov Test for Goodness of Fit." *Journal of the American Statistical Association* 46 (253): 68–78. https://doi.org/10.1080/01621459.1951.10500769.

Nguyen, Tuan Minh, Adib Shafi, Tin Nguyen, and Sorin Draghici. 2019. "Identifying Significantly Impacted Pathways: A Comprehensive Review and Assessment." *Genome Biology* 20 (1): 1–15. https://doi.org/10.1186/s13059-019-1790-4.

Subramanian, A., P. Tamayo, V. K. Mootha, S. Mukherjee, B. L. Ebert, M. A. Gillette, A. Paulovich, et al. 2005. "Gene Set Enrichment Analysis: A Knowledge-Based Approach for Interpreting Genome-Wide Expression Profiles." *Proceedings of the National Academy of Sciences* 102 (43): 15545–50. https://doi.org/10.1073/pnas.0506580102.

Tarca, Adi Laurentiu, Sorin Draghici, Gaurav Bhatti, and Roberto Romero. 2012. "Down-Weighting Overlapping Genes Improves Gene Set Analysis." *BMC Bioinformatics* 13 (1): 136. https://doi.org/10.1186/1471-2105-13-136.

Tarca, Adi Laurentiu, Sorin Draghici, Purvesh Khatri, Sonia S. Hassan, Pooja Mittal, Jung-sun Kim, Chong Jai Kim, Juan Pedro Kusanovic, and Roberto Romero. 2009. "A Novel Signaling Pathway Impact Analysis." *Bioinformatics* 25 (1): 75–82. https://doi.org/10.1093/bioinformatics/btn577.

Voichita, Calin, Sahar Ansari, and Sorin Draghici. 2021. "ROntoTools : The R Onto-Tools Suite."

Wang, Jing, Dexter Duncan, Zhiao Shi, and Bing Zhang. 2013. "WEB-Based GEne SeT AnaLysis Toolkit (WebGestalt): Update 2013." *Nucleic Acids Research* 41 (W1): W77–83. https://doi.org/10.1093/nar/gkt439.

Wilcoxon, Frank. 1992. "Individual Comparisons by Ranking Methods." In , 196–202. https://doi.org/10.1007/978-1-4612-4380-9_16.

Wu, Di, and Gordon K. Smyth. 2012. "Camera: A Competitive Gene Set Test Accounting for Inter-Gene Correlation." *Nucleic Acids Research* 40 (17): e133–e133. https://doi.org/10.1093/nar/gks461.
